# Development of water-soluble prodrugs of the bisdioxopiperazine topoisomerase IIβ inhibitor ICRF-193 as potential cardioprotective agents against anthracycline cardiotoxicity

**DOI:** 10.1038/s41598-021-83688-x

**Published:** 2021-02-24

**Authors:** Hana Bavlovič Piskáčková, Hana Jansová, Jan Kubeš, Galina Karabanovich, Nela Váňová, Petra Kollárová-Brázdová, Iuliia Melnikova, Anna Jirkovská, Olga Lenčová-Popelová, Jaroslav Chládek, Jaroslav Roh, Tomáš Šimůnek, Martin Štěrba, Petra Štěrbová-Kovaříková

**Affiliations:** 1 Department of Pharmaceutical Chemistry and Pharmaceutical Analysis, Faculty of Pharmacy in Hradec Králové, Charles University, Akademika Heyrovského 1203, 500 05 Hradec Králové, Czech Republic; 2 Faculty of Medicine in Hradec Králové, Charles University, Šimkova 870, 500 03 Hradec Králové, Czech Republic

**Keywords:** Pharmaceutics, Drug discovery and development, Bioanalytical chemistry

## Abstract

The bisdioxopiperazine topoisomerase IIβ inhibitor ICRF-193 has been previously identified as a more potent analog of dexrazoxane (ICRF-187), a drug used in clinical practice against anthracycline cardiotoxicity. However, the poor aqueous solubility of ICRF-193 has precluded its further in vivo development as a cardioprotective agent. To overcome this issue, water-soluble prodrugs of ICRF-193 were prepared, their abilities to release ICRF-193 were investigated using a novel UHPLC-MS/MS assay, and their cytoprotective effects against anthracycline cardiotoxicity were tested in vitro in neonatal ventricular cardiomyocytes (NVCMs). Based on the obtained results, the bis(2-aminoacetoxymethyl)-type prodrug GK-667 was selected for advanced investigations due to its straightforward synthesis, sufficient solubility, low cytotoxicity and favorable ICRF-193 release. Upon administration of GK-667 to NVCMs, the released ICRF-193 penetrated well into the cells, reached sufficient intracellular concentrations and provided effective cytoprotection against anthracycline toxicity. The pharmacokinetics of the prodrug, ICRF-193 and its rings-opened metabolite was estimated in vivo after administration of GK-667 to rabbits. The plasma concentrations of ICRF-193 reached were found to be adequate to achieve cardioprotective effects in vivo. Hence, GK-667 was demonstrated to be a pharmaceutically acceptable prodrug of ICRF-193 and a promising drug candidate for further evaluation as a potential cardioprotectant against chronic anthracycline toxicity.

## Introduction

**Figure 1:**
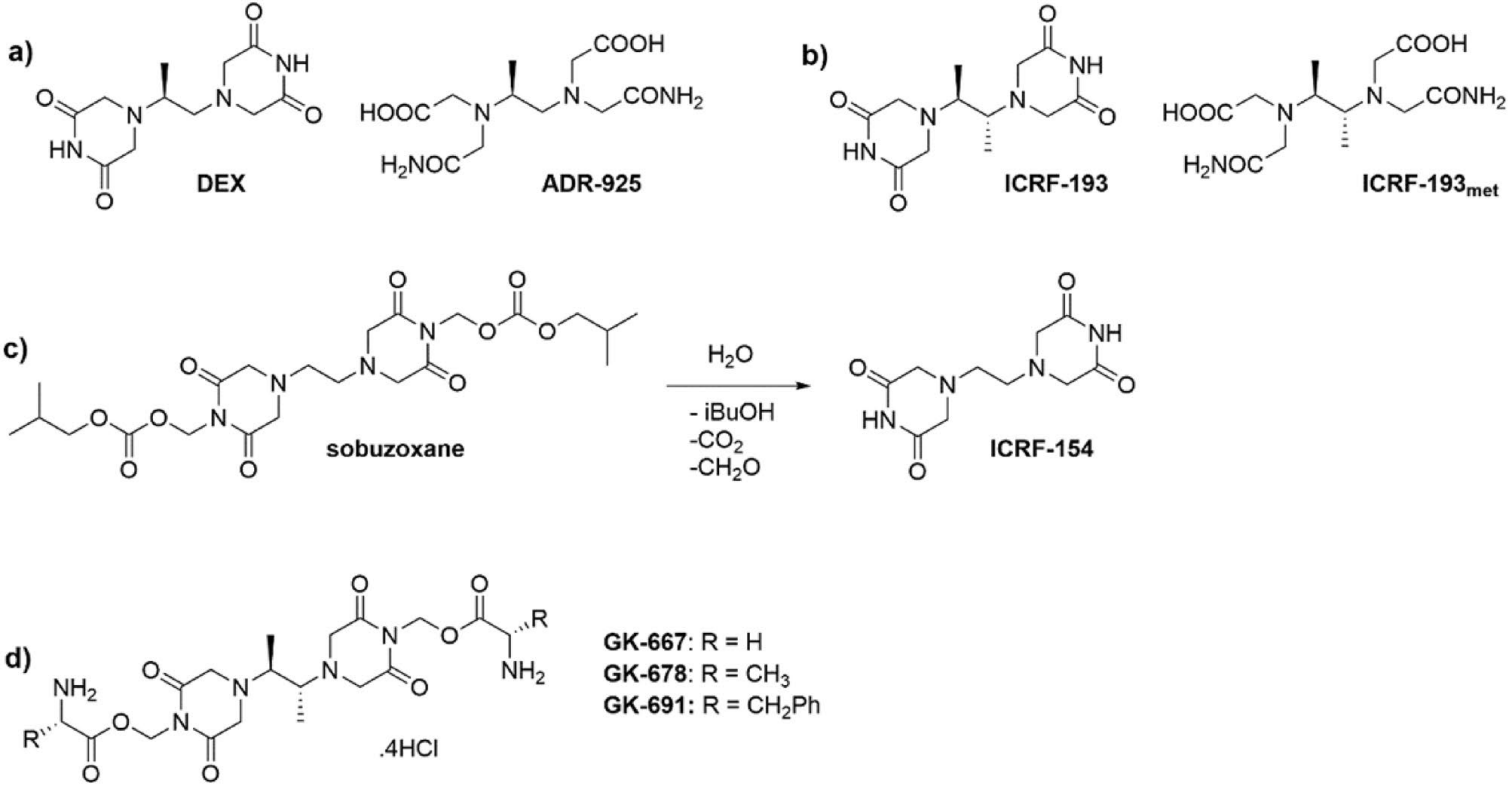
Chemical structures of bisdioxopiperazines, their metabolites and the prodrugs. Chemical structure of (**a**) dexrazoxane (DEX), its metabolite ADR-925 and (**b**) ICRF-193 and its proposed metabolite—ICRF-193_met_. (**c**) The activation pathway of sobuzoxane to ICRF-154. (**d**) General chemical structure of prepared prodrugs of ICRF-193.

Cardiotoxicity is one of the most serious adverse effects of anticancer chemotherapy and has a high impact on the morbidity, mortality and quality of life of long-term cancer survivors^1^. Severe cumulative dose-dependent cardiotoxicity is associated with anthracyclines (ANTs), which are among the most effective and widely used components of current chemotherapeutic protocols^1,2^. The most feared are chronic forms of cardiotoxicity, which can result in cardiomyopathy and congestive heart failure^3,4^. To date, dexrazoxane (DEX, ICRF-187, Fig. 1a) is the only drug approved for the prevention of ANT-induced cardiac damage in clinical practice^3,4^.

The cardioprotective potential of DEX (or its racemic form razoxane) has been recognized in studies that accompanied the investigation of the anticancer activity of bisdioxopiperazine derivatives. However, in contrast to their anticancer effects, the structure-activity relationships of the cardioprotective effects remain poorly characterized, and the corresponding molecular mechanisms responsible for effective cardioprotection are still the subject of discussion^4^. Limited available data suggest a very tight structure-activity relationship, as even minor changes in the chemical structure may result in the complete loss of cardioprotection^5,6^. These slight changes can be clearly exemplified by the simple substitution of a hydrogen for a methyl group on either the imide nitrogen or on a single piperazine ring^6^. Similar effects have been shown for the single-carbon elongation of the aliphatic linker, which demonstrates deterioration of the cardioprotective effect^7^. Conversely, it has recently been shown that simple methylation of C2 of the aliphatic linker in the *meso* configuration (i.e., ICRF-193; Fig. 1b) is promising regarding its cardioprotective potential against ANT cardiotoxicity^8^.

DEX has long been believed to be a prodrug that penetrates cardiomyocytes, where it is hydrolyzed to its active metabolite ADR-925 (Fig. 1a). As an EDTA analog, ADR-925 is able to bind free iron ions and remove them from redox-active complexes with ANT, and thus it should prevent the production of reactive oxygen species and oxidative injury to the myocardium^9^. However, the validity of this widely accepted concept has been disputed over the last decade^10^. Several important studies have challenged this hypothesis either directly or indirectly. Zhang et al. demonstrated that genetic knockout of topoisomerase IIβ (TOP2B) in cardiomyocytes can make the heart resistant to chronic ANT cardiotoxicity^11^. Others have proposed that the interaction of the parent compound DEX with this enzyme may be crucial and correlates with cardioprotective effects^5–8,12–15^.

A previous structure-activity study identified the markedly higher potency of ICRF-193 than DEX in both a TOP2B inhibitory assay and cardioprotection against anthracycline cardiotoxicity in vitro^8^. However, the physicochemical properties of ICRF-193 deserve attention before proposing it as a potential lead compound or drug candidate. In particular, ICRF-193 suffers from very poor solubility in aqueous vehicles owing to its very symmetrical chemical structure. This is a significant obstacle for the further development of this compound, as it effectively precludes its administration in vivo as well as appropriate examination of the cardioprotective potential in more complex and clinically relevant animal model of chronic ANT cardiotoxicity.

One of the common approaches to increase the solubility of drugs is the preparation of pharmaceutically acceptable prodrugs. The prodrug concept has already been successfully exploited with the related bisdioxopiperazine compound ICRF-154, which also suffers from poor water solubility and low in vivo bioavailability. Its prodrug, sobuzoxane, has been developed and approved for clinical use in Japan as an anticancer agent (Perazolin)^16^. Sobuzoxane is activated in the body through the hydrolysis of the carbonate esters into active bisdioxopiperazine (Fig. 1c)^17^, which improves its solubility and oral bioavailability^18^. It can be hypothesized that a similar concept using a carboxylic acid ester could be employed for tuning the pharmaceutical properties of ICRF-193 without compromising its cardioprotective potential and acceptable cytotoxicity (Fig. 1d). Based on its similar structure to DEX, it is expected that ICRF-193 released from a prodrug will also be prone to hydrolytic opening of its bisdioxopiperazine rings to form its metal-chelating metabolite (ICRF-193_met_, Fig. 1b). However, with respect to the above-discussed data, this metabolite should be considered inactive due to its lack of ability to interact with TOP2B.

The aim of the present study was to (1) prepare and chemically characterize several water-soluble prodrugs of ICRF-193, (2) develop UHPLC-MS/MS assays for their analyses together with ICRF-193 and its metabolite (ICRF-193_met_), (3) characterize the release and metabolism of ICRF-193 from the prodrugs in vitro under conditions relevant to the cytoprotective assay and in rabbit plasma, (4) determine the cardioprotective potential of these prodrugs against ANT cardiotoxicity in vitro and compare the results to those of ICRF-193, and (5) describe the in vivo pharmacokinetics of the selected prodrug along with ICRF-193 and ICRF-193_met_ in rabbits and determine basic pharmacokinetic parameters.

## Results and discussion

### Synthesis and initial analytical assay for the evaluation of the ICRF-193 prodrugs

To improve the solubility of ICRF-193 and enable future in vivo examination of its cardioprotective properties against ANT cardiotoxicity, we prepared three prodrugs (GK-667, GK-678 and GK-691, Fig. 1d) utilizing a concept similar to the design of sobuzoxane^16^. The syntheses of the target prodrugs were performed according to a known three-step procedure with some modifications^19^. Target prodrugs GK-667, GK-678 and GK-691 were obtained in 34%, 37% and 17% overall yields, respectively. While the approximate solubility of ICRF-193 in distilled water at room temperature is ≤ 0.005 mg/mL, the prodrugs showed markedly better solubilities with approximate values of 20, 20 and 10 mg/mL for GK-667, GK-678 and GK-691, respectively. The resulting solutions remained physically stable for at least two hours at room temperature.

A novel UHPLC-MS/MS assay was developed for the analysis of all three prodrugs, ICRF-193 and its expected two rings-opened metabolite (ICRF-193_met_). For details of the method development, validation and representative chromatograms, see the Supplementary material (Fig. S1). This method was used to characterize and compare the release of ICRF-193 from the prodrugs in cell medium (DMEM) to mimic exposure of the cells under the conditions of the in vitro experiments.

### ICRF-193 release from the prodrugs during incubation in cell culture medium and its further hydrolysis into the rings-opened metabolite

**Figure 2:**
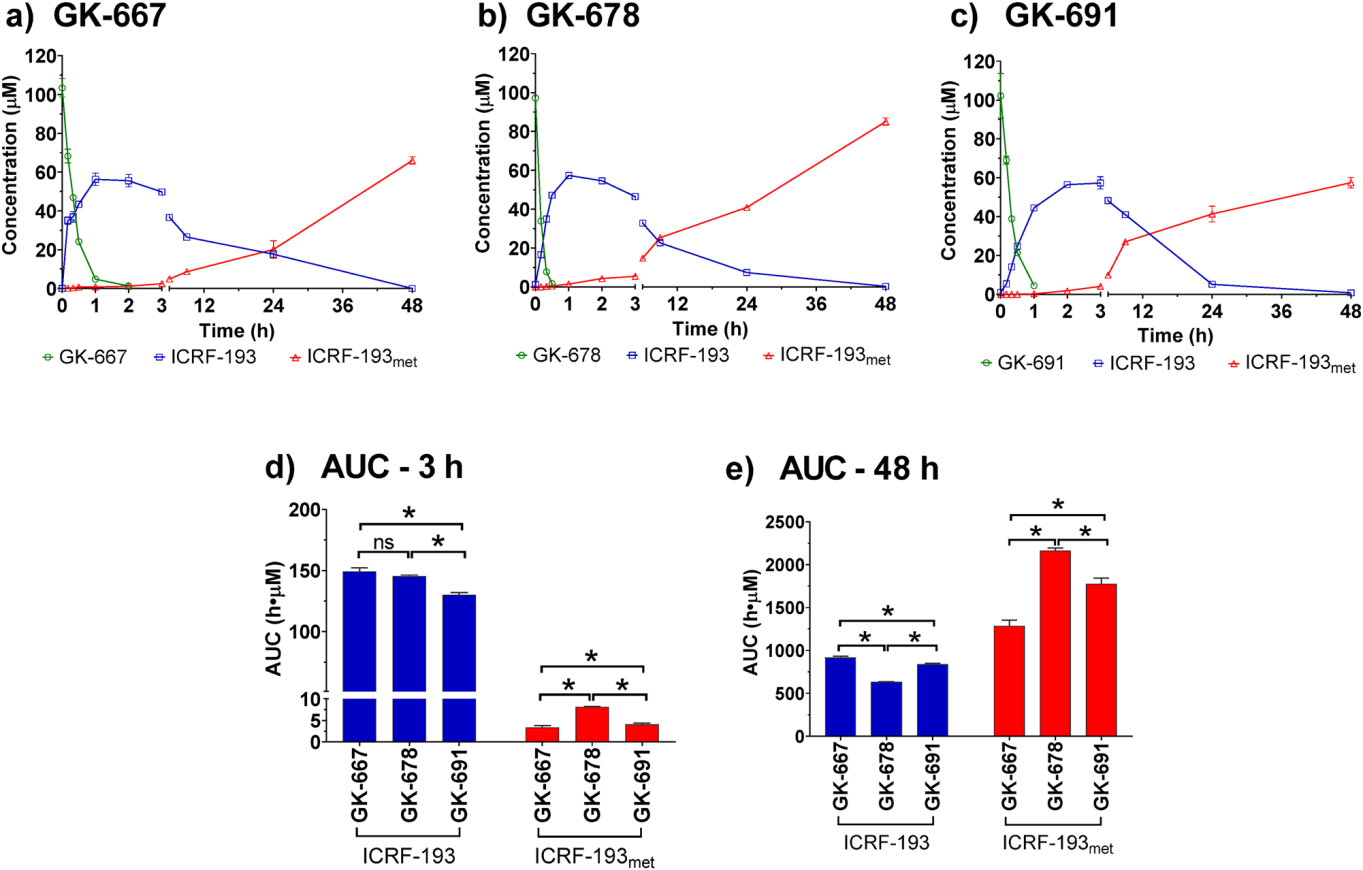
Activation of the prodrugs in cell medium—DMEM. Concentration time profiles of the prodrugs, ICRF-193 and ICRF-193_met_ obtained after incubation of (**a**) GK-667, (**b**) GK-678 and (**c**) GK-691 in DMEM (100 µM, 37 °C). Comparison of areas under the curve (AUC) of ICRF-193 and ICRF-193_met_ assayed at (**d**) 3 h and (**e**) 48 h of the prodrugs incubation in DMEM (100 µM, 37 °C). Data are presented as mean +/− SD (n = 4). Statistical significance was evaluated using one-way ANOVA and Holm-Sidak’s post-hoc test (*P* ≤ 0.05).

The prodrugs were incubated (100 µM) in DMEM (in thermomixer, at 350 rpm and 37 °C), and the release of ICRF-193 was examined using UHPLC-MS/MS. All of the prodrugs were found to undergo rapid decomposition in DMEM (Fig. 2), with GK-678 showing the fastest degradation (Fig. 2b). Prodrug degradation was accompanied by the release of ICRF-193, with the peak concentration of all the prodrugs of approximately 60 µM. However, the times at which the peak concentration of ICRF-193 were achieved were different for each prodrug. In all cases, the appearance of the metabolite ICRF-193_met_ was approximately inverse to the decrease in ICRF-193. For GK-678 and GK-691, equimolar amounts of ICRF-193 and ICRF-193_met_ were obtained after 9 and 12 h, respectively (Fig. 2b,c). Due to the slower decline in ICRF-193 concentrations in the case of GK-667, equimolar amounts of ICRF-193 and ICRF-193_met_ were achieved after 24 h (Fig. 2a).

To estimate the potential exposure of isolated rat neonatal ventricular cardiomyocytes (NVCMs) to active ICRF-193 under the in vitro experimental conditions, the areas under the curve (AUC_0–48 h_) of this compound and its metabolite ICRF-193_met_ were calculated (Fig. 2e). In addition, the potential exposures of the cells during the first 3 h (AUC_0–3 h_) were also calculated (Fig. 2d), as this represents the preincubation period in in vitro cardioprotective experiments, which is deemed essential for the induction of cardioprotection. As displayed in Fig. 2d,e, GK-667 and GK-678 showed no statistically significant differences in the AUC values of the ICRF-193 concentrations during the preincubation period (AUC_0–3 h_). However, the AUC values of the ICRF-193 concentrations during the whole experiment (AUC_0–48 h_) were significantly higher for GK-667 than GK-678. Importantly, GK-667 demonstrated the lowest conversion to the inactive metabolite ICRF-193_met_ from all prodrugs in both periods. These results together with the ability of GK-667 to quickly achieve a maximal ICRF-193 concentration favored this prodrug as the most suitable for further development.

### Cardioprotective effects of ICRF-193 and its prodrugs against ANT toxicity in an in vitro bioassay

The protective effects of ICRF-193 and the studied prodrugs against ANT toxicity were compared using the well-established in vitro cardioprotection bioassay. NVCMs were exposed to clinically relevant concentrations of daunorubicin (DAU, 1.2 µM) as the model ANT, and the cytotoxicity was determined as the LDH release after 48 h. First, we determined the cytotoxicity of the studied prodrugs alone towards NVCMs, and all prodrugs showed no or minimal toxicity (Fig. S2).

**Figure 3:**
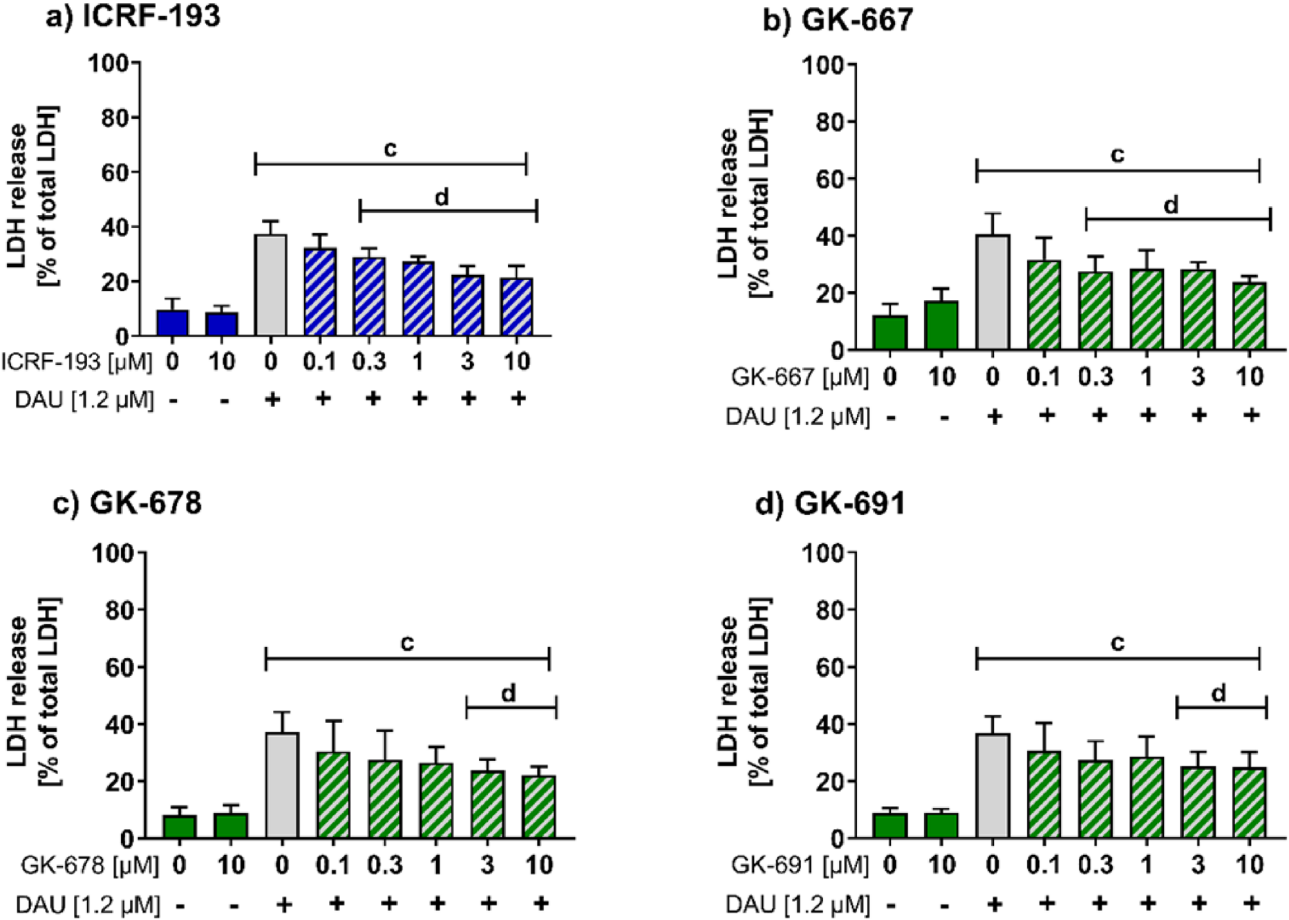
Cytoprotective effects of ICRF-193 and the prodrugs against anthracycline induced toxicity in neonatal ventricular cardiomyocytes. Neonatal ventricular cardiomyocytes were pre-incubated with (**a**) ICRF-193, (**b**) GK-667, (**c**) GK-678 or (**d**) GK-691 for 3 h, then DAU was added for another 3 h incubation followed by a 45 h period in medium without DAU or prodrug. The cytotoxicity was determined spectrophotometrically as lactate dehydrogenase (LDH) release in culture medium. Data are presented as the mean +/− SD of four independent experiments. Statistical significance was evaluated using one-way ANOVA and Holm-Sidak’s post-hoc test; c—compared to control, d—compared to DAU; *P* ≤ 0.05.

As shown in Fig. 3a–d, exposure of NVCMs to DAU induced significant cytotoxicity. ICRF-193 and all of the studied prodrugs displayed statistically significant and concentration-dependent protective effects against DAU toxicity. However, the studied agents differed in their concentrations required to provide these effects. ICRF-193 showed potent and concentration-dependent attenuation of DAU-induced toxicity from 0.3 µM (Fig. 3a). This confirmed the superior potency of this bisdioxopiperazine derivate compared to clinically used DEX, as the latter compound has consistently shown significant protective effects in this assay only at concentrations ≥ 10 µM^6^. Interestingly, GK-667 matched the results of ICRF-193 in the in vitro cytoprotection of NVCMs against DAU-induced toxicity. Both GK-691 and GK-678 showed the same significant protection at only 3 µM. The more potent cardioprotective effects of GK-667 compared with the other studied prodrugs may correspond with the rapid release of ICRF-193 and the slowest conversion to the TOP2B-inactive metabolite ICRF-193_met_ during incubation in DMEM, which promotes the highest exposure of the cells in both the preincubation and coincubation periods.

The results of this bioassay also supported the selection of GK-667 as the most suitable ICRF-193 prodrug for further detailed study, as it is nontoxic on its own and provides the most potent protective effects against DAU cardiotoxicity. Furthermore, the protective potency of this prodrug is the same as that of the active compound ICRF-193.

### UHPLC-MS/MS methods for advanced investigation of GK-667

The UHPLC-MS/MS assay developed for pilot analyses was further adopted for the determination of GK-667, ICRF-193 and ICRF-193_met_ in (1) DMEM and the corresponding buffer for comparison, (2) NVCMs to investigate the intracellular penetration and (3) rabbit plasma to simulate the in vitro release into the circulation after in vivo administration (method I). Although this method was capable of analyzing all in vitro samples, its sensitivity was insufficient for the determination of ICRF-193 and ICRF-193_met_ in plasma samples taken from the pilot in vivo pharmacokinetic study. Hence, method I was further modified to specifically achieve higher sensitivity for these key analytes in the in vivo pharmacokinetic study (method II). Both methods were fully validated (see Supplementary material), and representative chromatograms are shown in Fig. S3.

The process stability study showed that nearly 50% of GK-667 decomposed within 10 min when the samples were treated at room temperature (23 °C, Fig. S4a). This suggested that careful management of the preanalytical phase is necessary. Prodrug degradation could be significantly slowed when the samples were treated on ice, where less than 6% of GK-667 decomposed within 10 min (Fig. S4b). This is a sufficient length of time to allow for fast precipitation or sample dilution used in this study as sample pretreatment. These findings suggest that the biological samples where GK-667 is present require careful and immediate processing on ice to avoid artifacts and ensure appropriate validity of the results. In contrast, both ICRF-193 and ICRF-193_met_ are stable at laboratory temperature (Fig. S4a). We also found no significant degradation of ICRF-193 and ICRF-193_met_ in plasma when stored at -80 °C for 1 month (Table S6). These experiments indicated that plasma samples from later sampling intervals in the in vivo pharmacokinetic study where GK-667 is no longer present in significant amounts can be shock-frozen and stored at -80 °C for 1 month prior to analysis. After sample treatment (precipitation or dilution), all analytes (including GK-667) were stable prior to analysis in an autosampler set at 8 °C for up to 10 h (Table S7).

### Exposure of NVCMs to GK-667 and ICRF-193 under the in vitro cardioprotective bioassay conditions and intracellular penetration of the studied agents

An UHPLC-MS/MS assay (method I) was used to properly examine the exposure of the cells to the prodrug GK-667 and active compound ICRF-193 under in vitro cardioprotective bioassay conditions. The concentration (100 µM) of GK-667 and ICRF-193 was chosen considering the (1) low cytotoxicity of GK-667 observed in our toxicity study, (2) limit of quantification of the UHPLC-MS/MS assay and (3) possibility of direct comparison with previous studies with DEX.

**Figure 4:**
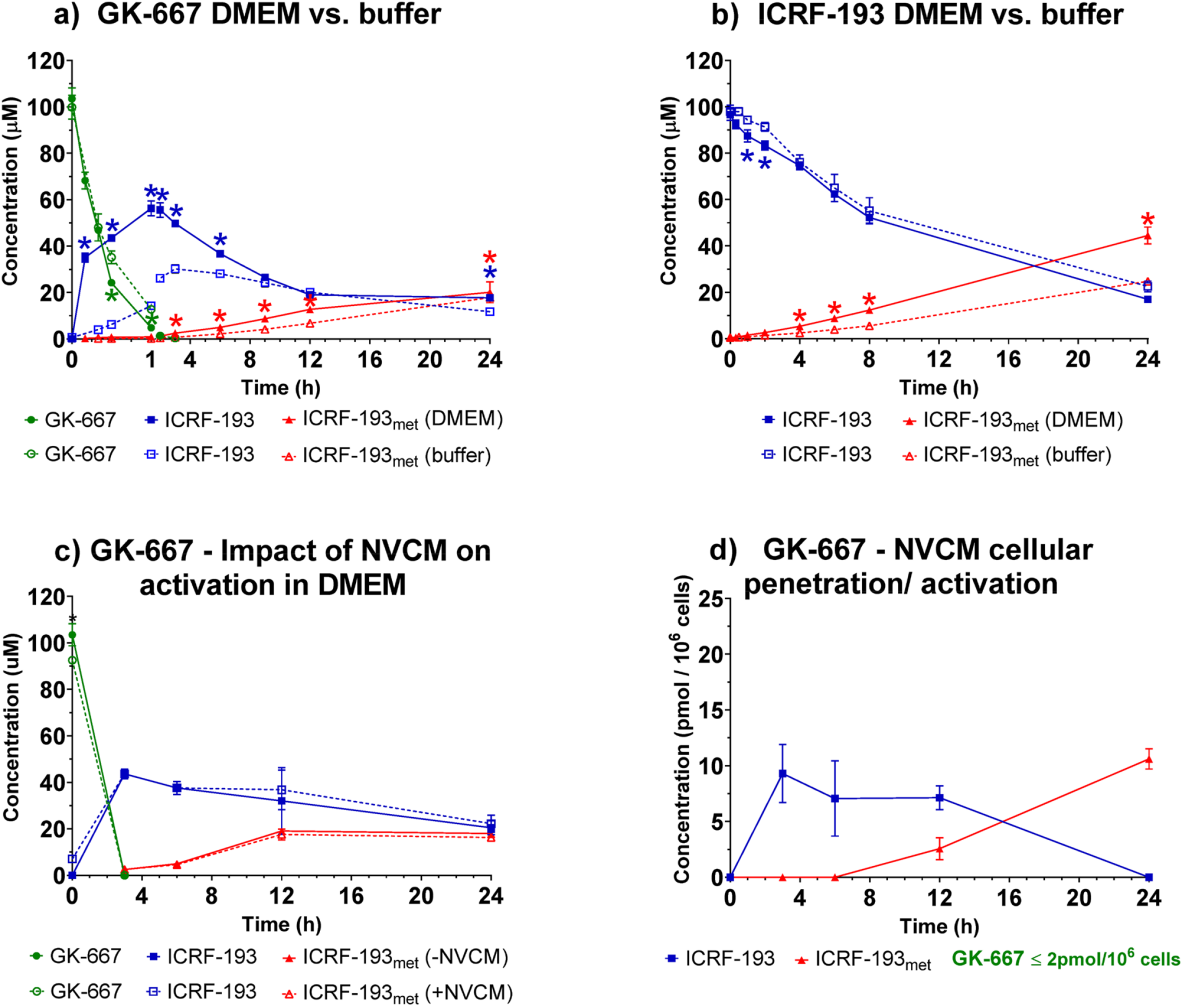
Activation of GK-667 and ICRF-193 in DMEM, the buffer and NVCMs. Concentration time profiles of the compounds after incubation of (**a**) GK-667 or (**b**) ICRF-193 (both 100 µM, 37 °C) in DMEM (solid symbols) and the buffer (open symbols). (**c**) Concentration time profile after incubation of GK-667 (100 µM, 37 °C) in DMEM with (open symbols) and without (solid symbols) neonatal ventricular cardiomyocytes (NVCMs). (**d**) Intracellular concentrations of the compounds after incubation of GK-667 (100 µM, 37 °C) with NVCMs. Data were collected from four independent experiments and are presented as the mean +/− SD. Statistical analysis was calculated in GraphPad Prism 8.4.3 using *t*-test correct for multiple comparisons using the Holm-Sidak method; *P* ≤ 0.005. *-compared to buffer.

GK-667 rapidly degraded in DMEM when kept without cells in an incubator (5% CO_2_, 37 °C) under the conditions of the in vitro experiments (Fig. 4a). The decrease in its concentration as well as the release of ICRF-193 were similar, as in the simulation experiments of these conditions in the thermomixer described above. A similar concentration profile for GK-667 was also observed when the same experiments were performed in saline buffer (containing NaCl, KCl, MgSO_4_, CaCl_2_, NaH_2_PO_4_, glucose, and HEPES, further named as buffer); statistically significant differences when compared with the results for the buffer were found only at 2 timepoints. Nevertheless, the amount of ICRF-193 assayed in the buffer was significantly lower than that assayed in DMEM (Fig. 4a). These data indicate that specific components of the medium (e.g., amino acids and glucose) marginally contribute to the decomposition of GK-667, but it is possible that they may accelerate an intermediate step of GK-667 activation into ICRF-193. A similar study was performed previously with sobuzoxane, which is activated to form bisdioxopiperazine ICRF-154^17^. In comparison with that of the carboxylate-type prodrug GK-667, decomposition of the carbonate-type prodrug sobuzoxane (100 µM) was much slower in DMEM under the same conditions^17^. The increase in ICRF-193 in the present experiment did not occur in an amount that was equimolar to the decrease in the concentration of the prodrug. At the later time intervals, the amount of rings-opened metabolite ICRF-193_met_ also did not occur in an amount that was equimolar to the decrease in the concentration of the parent compound ICRF-193. In addition to technical issues with the limited solubility of some of these compounds in aqueous environments, it should be pointed out that a significant part of the mass balance deficit could be due to several intermediate degradation products that were not determined in this study (e.g., hydroxymethyl-ICRF-193 or single ring-opened intermediates of ICRF-193). Similar findings have been previously reported for sobuzoxane^17^.

After incubation of ICRF-193 in DMEM or the related buffer, the compound degraded into its metabolite ICRF-193_met_, as expected. The rate of degradation of ICRF-193 was similar in both media, with no significant differences except for at 2 sampling intervals (Fig. 4b). The increases in the concentrations of ICRF-193_met_ were significantly slower in the buffer than in DMEM from the 4th hour of incubation onward (Fig. 4b). The mass balance deficit that can be seen in the present data may be related to single ring-opened metabolites analogous to intermediates B/C in the case of DEX^9^. Similar findings were previously observed after incubation of DEX in DMEM or buffer under similar conditions^20^.

To assess whether the metabolic capacity of NVCMs can contribute to the activation of GK-667 to ICRF-193 or its further hydrolysis to ICRF-193_met_ in cell medium, the same experiments were performed with and without the presence of NVCMs. As shown in Fig. 4c, the presence of cells had no significant effect on the metabolism of either GK-667 or ICRF-193. This finding is in line with that observed for DEX^20^.

In the next step, we determined the intracellular concentrations of the studied compounds after incubation of the cells with GK-667 (Fig. 4d). No parent prodrug was detected inside the cells, which corresponds to the fast degradation of the prodrug in DMEM. In contrast, ICRF-193 released from GK-667 showed relatively fast penetration into NVCMs, with the highest intracellular concentration (approximately 10 pmol/10^6^ cells) observed at the first sampling interval (3 h). The peak ICRF-193 concentration found in this study corresponds to approximately 50% of what was previously determined for DEX^20^. The markedly higher potency of ICRF-193 in cardioprotective assays reported herein and previously^8^ as well as the higher potency in the TOP2B inhibition assay^8^ may explain why these lower intracellular concentrations are sufficient for the protection of NVCMs. The intracellular profile of the inactive metabolite ICRF-193_met_ detected in this study (Fig. 4d) basically resembled that of ADR-925 after incubation of NVCMs with DEX^20^.

### In vitro study of ICRF-193 release from GK-667 in rabbit plasma and its further metabolism

The bioactivation of GK-667 (10 and 100 µM) into ICRF-193 was studied in rabbit plasma to simulate the in vitro fate of the prodrug after its administration to the circulation in vivo. The higher concentration of used GK-667 allowed for direct comparison with the other experiments presented above and the previously published study with DEX, while the lower concentration (10 µM) reflected the expected plasmatic concentrations after its in vivo administration.

**Figure 5:**
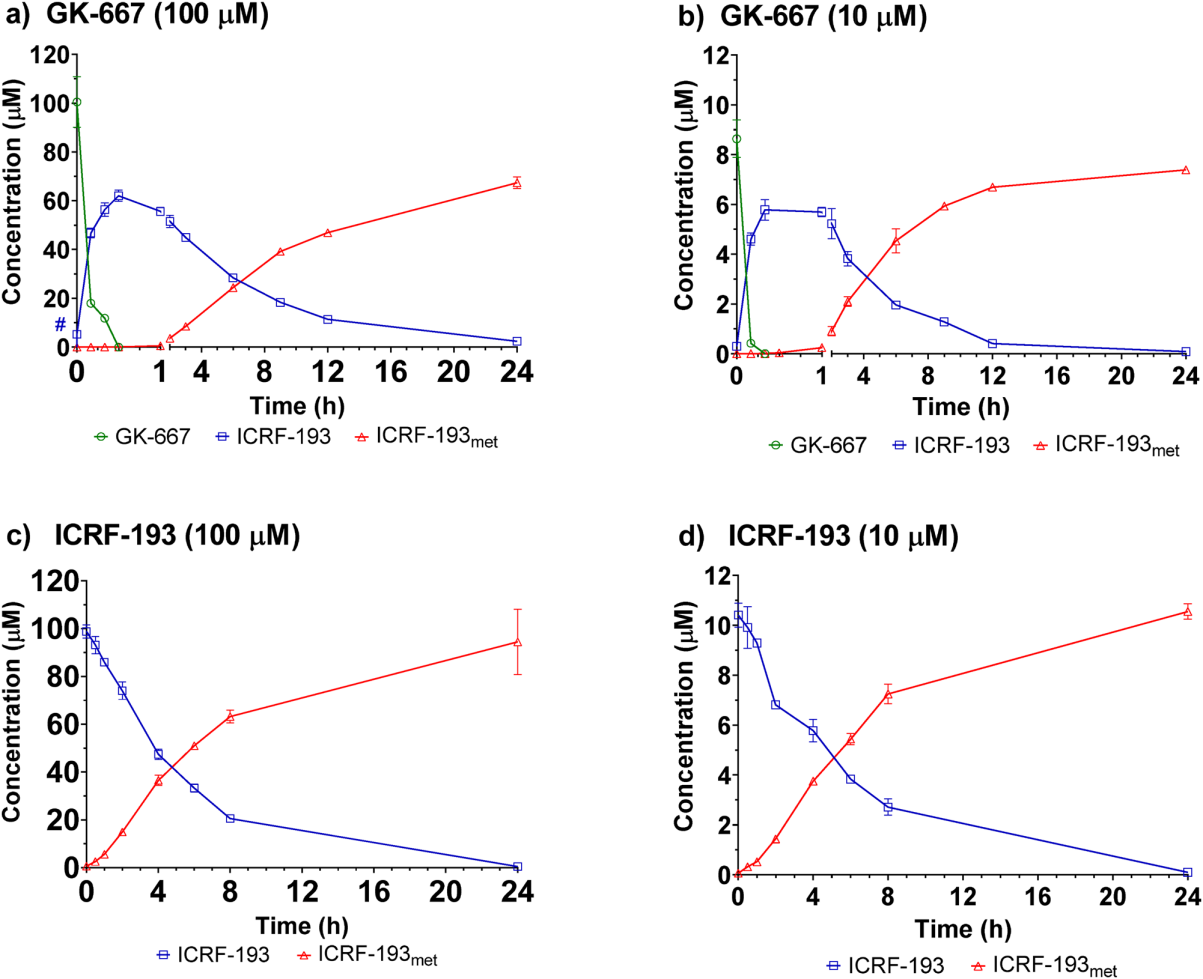
Activation of GK-667 and ICRF-193 in plasma in vitro. Concentration time profiles of the compounds after incubation of (**a**,**b**) GK-667 or (**c**,**d**) ICRF-193 in rabbit plasma in vitro (37 °C). The compounds were incubated in either (**a**,**c**) 100 or (**b**,**d**) 10 µM. Data are expressed as the mean +/− SD of four independent experiments.

GK-667 is rapidly metabolized in plasma (Fig. 5a). A decrease in the prodrug concentration was accompanied by the release of ICRF-193 at a peak concentration (65 µM) 30 min after initiation of the experiment. ICRF-193 was further converted into its metabolite. After 24 h, the amount of metabolite present was at an equimolar amount to the maximal concentration of ICRF-193. Both the decrease in GK-667 and the conversion of ICRF-193 to its metabolite were significantly faster (*P* ≤ 0.005, *t*-test correct for multiple comparisons) in plasma than in buffer and DMEM at all but 2 timepoints. This indicates that plasma components accelerate these processes. The first step may be catalyzed by plasma esterases, as was observed previously for sobuzoxane^16,17^. However, the second step, i.e., ICRF-193 conversion to ICRF-193_met_, is more likely accelerated nonenzymatically, e.g., by the presence of ions (e.g., Fe^2+^ and Ca^2+^), which was previously described for DEX^21,22^. Similar behaviors were observed for GK-667 in rabbit plasma at both concentrations (Fig. 5a,b), suggesting that activation in the studied range is concentration-independent.

To gain insights into the stability of ICRF-193 in plasma, this compound was incubated alone under the same conditions. As shown in Fig. 5c,d, the concentration of ICRF-193 gradually decreased in plasma and was accompanied by an increase in ICRF-193_met_ concentrations. In contrast to the incubation of ICRF-193 in DMEM and buffer, the mass balance comparison in plasma indicated the presence of only a minor amount of single ring-opened intermediates of ICRF-193 in this experiment. Both the degradation of ICRF-193 and increase in the metabolite in plasma are similar to what was previously observed with DEX and ICRF-154^17,20^. For GK-667 bioactivation, conversion of ICRF-193 to ICRF-193_met_ was also found to be concentration-independent.

### In vivo pharmacokinetic study in rabbits

The next step was to characterize the pharmacokinetics of the prodrug, active compound ICRF-193 and its rings-opened metabolite ICRF-193_met_ in vivo in an animal model to determine whether the plasma concentrations of ICRF-193 and the corresponding heart exposure are promising enough to justify future investigations of the cardioprotective effects of this prodrug in a chronic in vivo animal model.

The particular dose of the GK-667 salt (5 mg/kg, i.e., 3.78 mg/kg GK-667 free base containing 2.34 mg of ICRF-193) was selected for the first in vivo experiment based on the recommended ratio of DEX to ANT in the clinical setting^23,24^ and the in vitro potency ratio difference between ICRF-193 and DEX^8^. This dose of the prodrug was easily soluble in a commercially available saline solution (sodium chloride 0.9%, B-Braun, Germany) at a concentration of 5 mg/mL, which is convenient for administration to rabbits as an experimental animal model and could also be used for future pharmacodynamics experiments. This prodrug solution was found to be physically and chemically stable at laboratory temperature (20 °C) for at least 1 h (the concentrations of GK-667 did not fall below 95% of the initial amount, and no precipitate was visible in the solution).

#### Plasma concentration-time profiles

**Figure 6:**
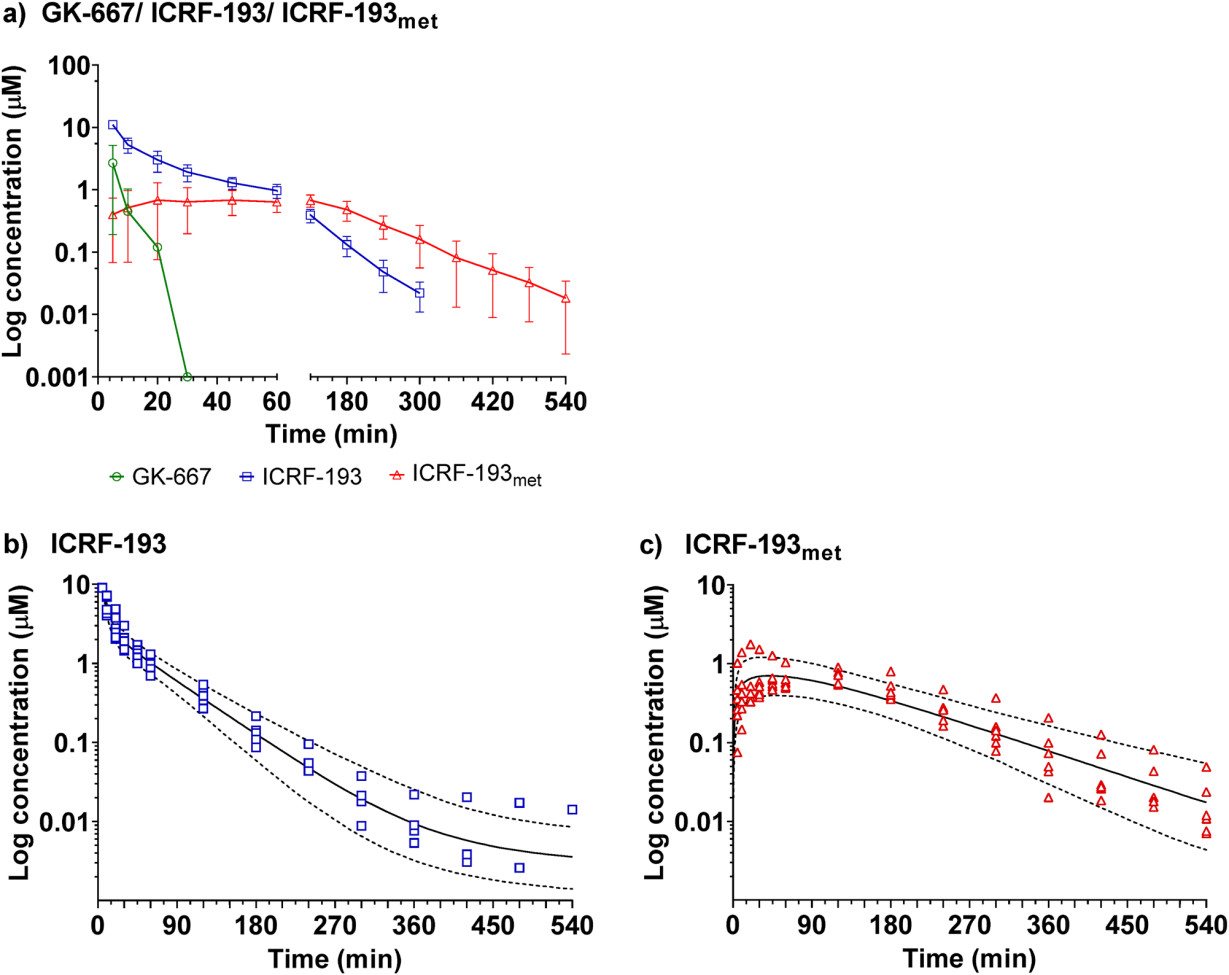
Plasma concentration profiles of the prodrug, ICRF-193 and ICRF-193_met_ after intravenous administration of GK-667 to rabbits. (**a**) Concentration time profiles of all compounds in plasma obtained after administration of GK-667 (5 mg/kg, *i.v.*) to rabbits (n = 6). Goodness-of-fit graphs of the population model of (**b**) ICRF-193 and (**c**) ICRF-193_met_. The solid and dashed lines represent the predicted median plasma concentrations and the 90% prediction intervals.

The plasma concentration-time profiles of GK-667, ICRF-193 and ICRF-193_met_ after administration of GK-667 to rabbits (n = 6) as a slow (2 min) *i.v.* bolus are presented in Fig. 6a. Peak concentrations of GK-667 were determined in plasma at the first sampling interval (5 min), and the subsequent plasma disappearance rate of GK-667 was very fast. The mean plasma concentration of the prodrug dropped from 2.7 to 0.4 µM between the 5th and 10th min, and after another 10 min, the concentrations were below the LLOQ (0.1 µM) in all but one animal. The highest concentrations of the active metabolite ICRF-193 were also observed in individual profiles at the first sampling interval (5 min). Moreover, ICRF-193 was the prevailing compound in plasma at this timepoint, as judged by both the mean (range) c_max_ of 11.0 (9.0–12.5) µM and ICRF-193-to-GK-667 concentration ratio of 7.2 (range: 1.5–14.8). A triphasic decline was apparent on the concentration-time curve of ICRF-193. Due to the relatively rapid decline in the ICRF-193 concentration, a submicromolar level was reached after 1 h of study initiation. At 4 h post injection, the concentration of ICRF-193 was above the LLOQ in all animals but already less than 1% of the corresponding c_max_, while one hour later, only two rabbits had concentrations higher than the LLOQ (0.01 µM). The metabolite ICRF-193_met_ was detected in the first interval in all rabbits, but its concentrations were more than one order of magnitude lower than those of ICRF-193. The concentration of ICRF-193_met_ peaked at the median t_max_ of 2 h (the range of individual t_max_ values was from 20 min to 2 h). The mean (range) c_max_ of 0.8 (0.5–1.8) µM was markedly lower than that of the parent compound ICRF-193.

The maximal concentration of ICRF-193 in plasma (approximately 11 µM) was approximately 30-fold higher than the lowest effective concentration in the in vitro cardioprotective assay (0.3 µM), which suggests a reasonable chance to induce cardioprotective effects in vivo. Furthermore, if ANTs are administered as recommended for DEX, i.e., 30 min after the cardioprotectant, the concentration of ICRF-193 at this time point (approximately 2 µM) is still well within the in vitro effective concentration range and remained in this range for approximately 3 h. This corresponds to the length of the preincubation period of NVCMs with the prodrug in our in vitro experiments^5^. However, future pharmacodynamic investigations utilizing in vivo models of chronic ANT cardiotoxicity are necessary to directly establish the cardioprotective potential of GK-667. Analogously, as with the administration of DEX to rabbits^20^, the metabolite ICRF-193_met_ could be detected in plasma 5 min after dosing, and its profile showed a plateau at approximately 30 min, which was followed by a gradual decline.

#### Noncompartmental pharmacokinetic analysis

Given the fast and complete conversion of GK-667 to ICRF-193 in rabbit plasma *in vitro* and the initial concentration observed in vivo, it was justifiable to make two assumptions. First, we assumed that the amount of GK-667 converted to ICRF-193 equals the injected dose, i.e., the absolute bioavailability of ICRF-193 is complete (F = 1). Second, we assumed that the mean input time for ICRF-193 is much less than the mean residence time after i.v. administration (MRT i.v.).

The pharmacokinetic parameters calculated by noncompartmental analysis (NCA) are summarized in Table 1. Blood sampling over 9 h was adequate for the description of the plasma pharmacokinetics of ICRF-193 and ICRF-193_met_. The extrapolated portions of the AUCs and AUMCs were less than 5% and 17%, respectively. The most noteworthy differences between the present NCA results and the pharmacokinetic characteristics of DEX in the same species are a shorter elimination half-life (0.8 vs 2.0 h) and a higher clearance of ICRF-193 (1.9 vs. 0.3 L/h/kg), which correspond to the faster disappearance of ICRF-193 from plasma compared to DEX. Furthermore, the half-life of elimination of the rings-opened metabolite of ICRF-193 (ICRF-193_met_) also appeared to be shorter than that of ADR-925 formed in vivo from DEX (1.4 vs. 2.9 h). Thus, although NCA identified particular differences between DEX and ICRF-193, these differences should not be an obstacle for future evaluation of the cardioprotective effects of ICRF-193 *in* *vivo* because the plasma pharmacokinetics of GK-667 provides sufficient myocardial exposure to justify the pharmacodynamic experiments.

**Table 1:** Summary of pharmacokinetic parameters of GK-667, ICRF-193 and ICRF-193_met_ in rabbits determined by non-compartmental analysis.

	C_max_ (µM)	T_max_ (min)	AUC_last_ (h∙µM)	AUC_tot_ (h∙µM)	AUMC_last_ (h^2^∙µM)	AUMC_tot_ (h^2^∙µM)	λ_z_ (h^−1^)	t_1/2_ (h)	MRT (h)	CL (L h^−1^ kg^−1^)	V_z_ (L kg^−1^)	V_ss_ (L kg^−1^)
GK-667	2.7	5	–	–	–	–	–	–	–	–	–	–
	(0.61–7.4)	(5–5)	–	–	–	–	–	–	–	–	–	–
ICRF-193	11.0	5	4.4	4.4	2.9	3.0	0.94	0.78	0.69	1.9	2.2	1.3
	(9.0–12.5)	(5–5)	(3.5–5.3)	(3.6–5.3)	(1.8–4.0)	(2.1–4.1)	(0.55–1.1)	(0.66–1.3)	(0.59–0.92)	(1.6–2.3)	(1.5–3.4)	(1.0–1.7)
ICRF-193_met_	0.83	120	2.6	2.7	6.5	7.1	0.52	1.4	2.6	–	–	–
	(0.54–1.8)	(20–120)	(1.9–3.6)	(1.9–3.8)	(4.3–11.4)	(4.5–13.5)	(0.36–0.63)	(1.1–1.9)	(1.9–3.6)	–	–	–

*C*_*max*_ observed maximum plasma concentration, *T*_*max*_ time to reach maximum plasma concentration, *AUC*_*last*_ area under the concentration-time curve from zero up to the last quantifiable concentration, *AUC*_*tot*_ area under the concentration-time curve from zero up to ∞ with extrapolation of the terminal phase, *AUMC*_*last*_ area under the first moment of the concentration-time curve from zero to the last quantifiable concentration, *AUMC* area under the first moment of the concentration-time curve from zero up to ∞ with extrapolation of the terminal phase, *λ*_*z*_ terminal rate constant of elimination, *t*_*1/2*_ terminal half-life, *MRT* mean residence time, *CL* total clearance, *V*_*z*_ apparent volume of distribution during terminal phase of elimination, *V*_*ss*_ apparent volume of distribution at equilibrium.

#### Population pharmacokinetic analysis

Assuming an instant input of ICRF-193 solely into the central compartment (V_1_) and complete conversion of the administered dose of GK-667 into ICRF-193, the disposition of ICRF-193 in plasma was best described by a three-compartment open model with first-order elimination from the central compartment. In the joint model for ICRF-193 and ICRF-193_met_, formation of the metabolite was modeled as a first-order process (Fig. S5), and one volume of distribution for the metabolite (V_4_) was added to three volumes for ICRF-193 (V_1_, V_2_, and V_3_). As no data were available on the separate administration of ICRF-193_met_ due to its poor solubility, the formation clearance of the metabolite (CL_met_) was estimated assuming that the a priori population value of V_4_ equals the distribution volume of ADR-925 (a structurally very similar metabolite of DEX) observed in our former rabbit study^20^.

All model parameters of ICRF-193 and ICRF-193_met_ could not be simultaneously estimated by stochastic approximation expectation maximization (SAEM) in one step. Instead, population medians and between-subject standard deviations were first estimated for the volume (V_1_, V_2_ and V_3_) and clearance parameters (CL_tot_) for the parent molecule using the concentrations of ICRF-193. In the second step, the ICRF-193_met_ concentration data were added, median estimates for the parameters of ICRF-193 from the first step were used as initial estimates, and their standard deviations were fixed. A maximum likelihood estimation was used to calculate the parameters CL_met_, CL_other_, V_4_, and CL_ICRF-193met_. For the remaining parameters of the parent-metabolite model (Fig. S5), a maximum a posteriori (MAP) estimation was adopted. The residual error in the population kinetic analysis was best described with a proportional model.

Population estimates of the parameters and between-subject variability (standard deviation) are presented in Table 2. Considering the small size of the study (n = 6) and the absence of data on the excretion of the studied compounds, the median values of the pharmacokinetic characteristics were estimated with good precision, except for volumes V_3_ and V_4_ and the elimination clearance of the metabolite CL_ICRF-193met_. Estimates of the between-animal variability were less precise. The simulated median concentration and the 90% confidence interval provide good evidence of the adequate predictive performance of the model for the assayed concentrations of ICRF-193 and ICRF-193_met_ (Fig. 6b,c). The reliability of the model was further supported by scatter plots of the observed concentrations vs. individual predicted concentrations (Fig. S6).

**Table 2:** Pharmacokinetic parameters of ICRF-193 and ICRF-193_met_ in rabbits determined by population analysis.

	V_1_ (L kg^−1^)	V_2_ (L kg^−1^)	V_3_ (L kg^−1^)	Q_12_ (L h^−1^ kg^−1^)	Q_13_ (L h^−1^ kg^−1^)	CL_tot_ (L h^−1^ kg^−1^)	CL_met_ (L h^−1^ kg^−1^)	CL_other_ (L h^−1^ kg^−1^)	V_4_ (L∙kg^−1^)	CL_ICRF-193met_ (L∙h^−1^∙kg^−1^)
**ICRF-193**										
Median	0.36	0.67	0.98	1.4	0.035	1.7	0.081	1.6	na	na
Median %RSE	15	13	93	16	36	5.0	32	6.4	na	na
ISV	30	24	26	32	72	10	27	10	na	na
ISV %RSE	40	36	107	47	67	35	42	35	na	na
**ICRF-193**_**met**_										
Median	na	na	na	na	na	na	na	na	0.31	0.17
Median %RSE	na	na	na	na	na	na	na	na	69	66
ISV	na	na	na	na	na	na	na	na	25	6.5
ISV %RSE	na	na	na	na	na	na	na	na	35	150

*V*_*1*_*, V*_*2*_* and V*_*3*_ distribution volumes of central and peripheral compartments for ICRF-193, *V*_*4*_ distribution volume for ICRF-193_met_, *CL*_*met*_ formation clearance of ICRF-193_met_ from ICRF-193, *CL*_*other*_ the sum of clearances of ICRF-193 via other elimination pathways than metabolism to ICRF-193_met_, *Q*_*12*_* and Q*_*13*_ inter-compartmental clearances, *CL*_*ICRF-193met*_ the total clearance of ICRF-193_met_. The estimates of clearances and volumes of distribution were obtained assuming F = 1; *ISV* inter-subject variability expressed as the percent CV, *na* not applicable, *%RSE* percent relative standard error.

## Conclusion

In this study, we confirmed the previous finding that DEX analog ICRF-193 is a potent cardioprotectant in vitro against ANT toxicity with significant protection of NVCMs observed from submicromolar concentrations of the drug. As the poor water solubility of this compound has precluded its in vivo administration, three prodrugs of ICRF-193 with water solubilities improved by several orders of magnitude were prepared and chemically characterized. Based on the effective release of ICRF-193, low toxicity and potent cytoprotective effects against ANT cardiotoxicity in NVCMs, GK-667 was selected for further testing. Using a UHPLC-MS/MS assay, we found that ICRF-193 is promptly released from GK-667 in cell culture medium and the related buffer under conditions relevant to the in vitro cardioprotective assay. The released ICRF-193 easily penetrated into NVCMs, reaching intracellular concentrations sufficient to induce cytoprotective effects against ANT toxicity. Rapid release of ICRF-193 from GK-667 was also found in rabbit plasma in vitro. Furthermore, GK-667 was dissolved in saline and administered to rabbits in vivo, and the pharmacokinetics of GK-667, ICRF-193 and the rings-opened metabolite were described. The plasma concentration-time profile of ICRF-193 was found to be adequate to achieve the potential cardioprotective effects under these in vivo conditions based on comparison with the effective concentrations in the in vitro cardioprotective assay. Thus, GK-667 was identified as a pharmaceutically acceptable prodrug of ICRF-193 and a promising drug candidate for further comprehensive in vivo examinations as a cardioprotective agent against chronic ANT toxicity.

## Materials and methods

### Chemicals and materials

ICRF-193 was commercially available (Merck, Germany). All prodrugs (GK-667, GK-678 and GK-691), ICRF-193, ICRF-193_met_ and internal standards (I.S._(A)_ for ICRF-193_met_ and I.S._(B)_ for both ICRF-193 and the prodrugs) were prepared as described below or in the Supplementary material. Preparation of I.S._(B)_ have been already described^6^. All compounds were characterized using NMR (Varian VNMR S500 spectrometer; Varian, CA, USA). The purity of the synthetized prodrugs was proven by NMR, MS and UHPLC-MS (Figs. S8–S10). All chemicals and materials are specified in the Supplementary material.

### Synthesis and characterization of the prodrugs

#### Synthesis of GK-667 (meso-2,3-bis(4-(2-aminoacetoxymethyl)-3,5-dioxopiperazin-1-yl)butane tetrahydrochloride)

A solution of *meso*-2,3-bis(4-(2-(*tert*-butoxycarbonylamino)acetoxymethyl)-3,5-dioxopiperazin-1-yl)butane (0.2 g, 0.3 mmol) in acetic acid (5 mL) was treated with an excess of HCl (g), and the reaction mixture stirred at rt for 2 h. Et_2_O (50 mL) was added, and the product was filtered, washed with Et_2_O and dried. Yield: 93% as a white solid; mp 156–159 °C. ^1^H NMR (500 MHz, DMSO-*d*_6_) δ 8.51 (t, *J* = 5.8 Hz, 6H), 5.75 (s, 4H), 3.79 (q, *J* = 5.7 Hz, 4H), 3.67 (d, *J* = 16.7 Hz, 4H), 3.59 (d, *J* = 16.8 Hz, 4H), 2.98 (s, 2H), 0.97 (d, *J* = 4.1 Hz, 6H). ^13^C NMR (126 MHz, DMSO-*d*_6_) δ 169.90, 166.92, 62.61, 59.17, 52.19, 9.75.

#### Synthesis of GK-678 (meso-2,3-bis(4-((S)-2-aminopropionyloxymethyl)-3,5-dioxopiperazin-1-yl)butane tetrahydrochloride)

A solution of *meso*-2,3-bis(4-((*S*)-2-(*tert*-butoxycarbonylamino)propionyloxymethyl)-3,5-dioxopiperazin-1-yl)butane (0.2 g, 0.3 mmol) in acetic acid (5 mL) was treated with an excess of HCl (g), and the reaction mixture stirred at rt for 2 h. Et_2_O (50 mL) was added, the product was filtered, washed with Et_2_O and dried. Yield: 86% as a white solid; mp 152–155 °C. ^1^H NMR (500 MHz, DMSO-*d*_6_) δ 8.65 (d, *J* = 5.5 Hz, 6H), 5.80 (dd, *J* = 9.8, 3.9 Hz, 2H), 5.71 (dd, *J* = 9.8, 4.5 Hz, 2H), 4.1–4.0 (m, 2H), 3.69 (d, *J* = 16.9 Hz, 4H), 3.61 (d, *J* = 16.9 Hz, 4H), 2.99 (s, 2H), 1.36 (d, *J* = 7.2 Hz, 6H), 1.01–0.95 (m, 6H). ^13^C NMR (126 MHz, DMSO-*d*_6_) δ 169.91, 169.25, 62.74, 59.24, 52.16, 47.85, 15.75, 9.81.

#### Synthesis of GK-691 (meso-2,3-bis(4-((S)-2-amino-3-phenylpropionyloxymethyl)-3,5-dioxopiperazin-1-yl)butane tetrahydrochloride)

A solution of *meso*-2,3-bis(4-((*S*)-2-(*tert*-butoxycarbonylamino)-3-phenylpropionyloxymethyl)-3,5-dioxopiperazin-1-yl)butane (0.25 g, 0.3 mmol) in acetic acid (5 mL) was treated with an excess of HCl (g), and the reaction mixture stirred at rt for 2 h. Et_2_O (50 mL) was added, the product was filtered, washed with Et_2_O and dried. Yield: 86% as a white solid; mp 150–153 °C. ^1^H NMR (500 MHz, DMSO-*d*_6_) δ 8.76 (s, 6H), 7.31–7.17 (m, 10H), 5.79 (dd, *J* = 9.8, 5.9 Hz, 2H), 5.62 (d, *J* = 9.8 Hz, 2H), 4.29–4.23 (m, 2H), 3.77–3.40 (m, 8H), 3.19–3.03 (m, 4H), 2.98 (s, 2H), 1.01–0.94 (m, 6H). ^13^C NMR (126 MHz, DMSO-*d*_6_) δ 169.80, 168.18, 168.15, 134.62, 134.55, 129.68, 128.73, 127.43, 62.74, 59.18, 59.11, 53.16, 53.11, 52.17, 35.73, 35.68, 9.73, 9.68.

### Chromatographic analyses

The preparation of stock and working solutions used for the UHPLC-MS/MS assay is described in the Supplementary materials.

#### Sample preparation

All samples were spiked with the internal standards. DMEM or buffer (100 μL) was diluted with an ice-cold methanol/water mixture (20:80, v/v), mixed and analyzed. Plasma (50 μL) was precipitated with ice-cold methanol (1:5, v/v), mixed (20 s) and centrifuged (10 min, 10,000 rpm, 4 °C). NVCM pellets (4.8 × 10^6^ cells) were precipitated with ice-cold methanol (250 μL), mixed (20 s), sonicated in a cold ultrasonic bath (75 s), mixed (30 s) and centrifuged (10 min, 10,000 rpm, 4 °C). The supernatants from the plasma and NVCM extractions were filtered (0.22 μm, PVDF) prior to analysis. For assay of the prodrugs, the samples were acidified with formic acid to a final concentration of 0.5%.

#### UHPLC-MS/MS methods

The samples were analyzed using two UHPLC instruments both coupled to a triple quadrupole mass spectrometer. A Nexera X2 with LCMS 8030 mass spectrometer, electrospray ionization and LabSolution software (Shimadzu, Japan) were utilized for the simultaneous assay of all prodrugs, ICRF-193 and ICRF-193_met_ in DMEM, buffer, NVCMs and rabbit plasma (method I). To increase sensitivity, an Agilent 1290 Infinity II LC with Triple Quad LC/MS (6400 series), a Jet Stream electrospray and a Mass Hunter software (Agilent, USA) were applied for determination of ICRF-193 and ICRF-193_met_ in plasma from the pharmacokinetic study (method II). Positive ionization mode was used in both methods. The analytical columns tested are characterized in the Supplementary materials.

The best separation was achieved with a Luna Omega Polar C18 column (100 × 2.1 mm, 1.6 μm, Phenomenex, USA) with the same type of guard column. Prior to the first analysis, the column was flushed with a mixture of 2 mM dipotassium EDTA solution and acetonitrile (80:20; v/v) (4 h using a flow rate of 0.25 mL/min) to remove metal ions from the chromatographic system.

A mixture of 1 mM ammonium formate and either acetonitrile (method I) or methanol (method II) was used in the following gradients: either 0.0–1.0 min (20% acetonitrile), 1.1–4.5 min (50–90% acetonitrile), 4.7–6.0 min (20% acetonitrile) or 0.0–1.0 min (20% methanol), 1.1–4.5 min (50–80% methanol), and 4.7–6.0 min (20% methanol) for methods I or II, respectively. The column and the autosampler thermostat were maintained at 30 °C and 8 °C, respectively. The flow rate of mobile phase was 0.25 mL/min. One (method I) or two (method II) μL of sample were injected onto the column. The chemical structures of the internal standards are shown in the Supplementary materials (Fig. S7). For details on the MS settings, see Tables S8 and S9.

Both methods were fully validated according to EMA guidelines for bioanalytical method validation^25^ within the concentration ranges that were sufficient for the analysis of all samples from both the in vitro studies and in vivo pharmacokinetic experiment. Method I was fully validated for the simultaneous determination of GK-667, ICRF-193 and ICRF-193_met_ in DMEM, buffer, rabbit plasma and NVCMs, and method II was fully validated for the determination of ICRF-193 and ICRF-193_met_ in rabbit plasma. For details, see the Supplementary material. The validation parameters are summarized in Tables S1–S5.

### In vitro study of ICRF-193 release from prodrugs in cell medium and rabbit plasma

First, all prodrugs prepared in this study were screened for their ability to release ICRF-193 into DMEM to estimate potential exposure to NVCMs during an in vitro cardioprotective assay. The prodrugs (100 μM) were incubated (37 °C) in DMEM in a thermomixer (Thermomixer Comfort, Eppendorf, Germany) under gentle mixing (350 rpm) for 48 h.

Further detailed studies were then performed with GK-667 as the selected prodrug and ICRF-193 as the active agent. Herein, incubations were performed in DMEM (100 μM) in a CelCulture Incubator (5% CO2, 37 °C, ESCO, USA) to mimic the conditions of the in vitro cytoprotective assay, and the results were compared to those from the saline buffer (116 mM NaCl, 5.3 mM KCl, 1.2 mM MgSO_4_, 1 mM CaCl_2_, 1.13 mM NaH_2_PO_4_, 5 mM glucose, and 20 mM HEPES, pH = 7.4).

In the next step, the release of ICRF-193 was studied at two concentrations (10 and 100 μM) in rabbit plasma as the relevant biological environment for the in vivo experiments. In these experiments, both GK-667 and ICRF-193 were incubated in rabbit plasma (37 °C) in a thermomixer (350 rpm).

From all of the above described experiments, samples were taken at predefined intervals, treated and immediately analyzed. All experiments were performed in four replicates, and the results are expressed as the mean ± SD.

### Impact of cultured NVCMs on ICRF-193 release and its metabolism in cell medium and corresponding intracellular concentrations of the studied agents

GK-667 (100 μM) was incubated (37 °C, 5% CO_2_, CelCulture Incubator) with NVCMs in the medium (DMEM/F12 supplemented with 1% P/S) for 24 h. Both DMEM and NVCMs were harvested at 3, 6, 12 and 24 h. Immediately after sampling, DMEM was treated and analyzed. Cardiomyocytes were scrapped, washed twice with PBS buffer (4 °C), and centrifuged (700 × *g*, 10 min, 4 °C). The redundant supernatant was discarded, and the pellet was immediately treated and analyzed. All experiments were performed in triplicate, and the results are expressed as the mean ± SD.

### In vitro assay of cardioprotective effects against ANT-induced toxicity

NVCMs were isolated in-house from 1- to 3-day-old Wistar rats as described previously^26^. The use of animals and experimental protocols were approved by Charles University, Faculty of Pharmacy Animal Welfare Committee. All experiments were performed in accordance with Directive 2010/63/EU on the Protection of Animals Used for Scientific Purposes and in compliance with Animal Research: Reporting of In Vivo Experiments (ARRIVE) guidelines. Newly isolated cardiomyocytes were left for 40 h to form a monolayer of spontaneously beating cardiomyocytes in seeding medium (DMEM/F12 supplemented with 10% HS, 5% FBS, 4% PYR and 1% P/S), which was then replaced with culture medium (DMEM/F12 supplemented with 5% FBS, 4% PYR and 1% P/S). The medium was replaced again after 24 h for new culture medium.

On the next day, the culture medium was replaced with exposure medium (DMEM/F12 supplemented with 1% P/S), in which solutions of ICRF-193 or each of the prodrugs in DMSO were added (DMSO alone was used as a control). After 3 h, DAU solution (1.2 µM) was added to the media. After another 3 h, the medium was replaced with new (drug-free) exposure medium, and the cardiomyocytes were then kept until the end of the experiment. In addition to cytoprotective properties, the toxicity of the studied agent was also examined; this experiment was performed as described above with only the DAU addition step omitted.

Forty-five hours after the last medium exchange, a sample of medium was taken from each well to assess lactate dehydrogenase (LDH) activity^27–29^. For details, see the Supplementary material.

### Data analysis

Data are presented as the mean ± SD unless stated otherwise. Statistical significance (P ˂ 0.05) was determined by one-way ANOVA followed by Holm-Sidak’s post hoc test or by *t*-test corrected for multiple comparisons according to the data characteristics. GraphPad Prism 8.4.3 software (GraphPad Software, CA, USA) was used for statistical analysis and graphical representation of the results.

### In vivo pharmacokinetic study of GK-667 in rabbits

The dose of GK-667 for the pharmacokinetic experiment was set at 5 mg/kg (i.e., 3.78 mg/kg GK-667 free base containing an equivalent of 2.34 mg of ICRF-193)^23^.

GK-667 was freshly dissolved in saline (5 mg/mL) before each administration. New Zealand White male rabbits (n = 6, ∼3.8 kg) received GK-667 (5 mg/kg) administered as a single slow (2 min) intravenous dose bolus to the ear marginal vein. Blood (∼1 mL) was sampled from veins on the contralateral ear at selected time points after drug administration (5 min to 9 h) into BD Vacutainers (BD Biosciences, Plymouth, UK) containing lithium heparin. Collected blood samples were immediately centrifuged (2 min, 3000 rpm, 4 °C), and plasma samples were harvested directly.

Plasma samples taken 0 to 20 min after GK-667 administration were immediately processed by the addition of formic acid and precipitation with ice-cold methanol. The supernatants were then analyzed for all analytes, including the intact prodrug. This was essential to ensure the appropriate determination of highly labile GK-667 at the first sampling intervals, and the whole procedure was validated for this purpose. Pilot experiments showed that in the plasma samples taken at later time intervals, the concentrations of the intact prodrugs were far below the LLOQ of the UHPLC-MS/MS assay, so that the procedure could be omitted as unsubstantiated. Thus, all following plasma samples were immediately shock-frozen in liquid nitrogen and stored at -80 °C until analysis.

For drug administration and blood sampling, light anesthesia was induced in rabbits with a mixture of ketamine (25 mg/kg, *i.m.)* and midazolam (2.5 mg/kg, *i.m*.); sedation was then maintained with midazolam when needed. The blood withdrawn from the animals was compensated with an appropriate volume of sterile saline. All in vivo experiments and procedures were approved and supervised by the Animal Welfare Committee of the Faculty of Medicine in Hradec Kralove (Charles University), and the experiments were performed in compliance with the ARRIVE guidelines.

#### Pharmacokinetic analysis of data from the in vivo experiments

Standard noncompartmental analysis was performed using Kinetica software (version 4.0, Thermo Fisher Scientific Inc., USA). The population parameter estimation for nonlinear mixed effect models was conducted using Monolix version 2019R2 software (Antony, France: Lixoft SAS, 2019, http://lixoft.com/products/monolix/). The stochastic approximation expectation maximization (SAEM) algorithm coupled with Markov Chain Monte Carlo (MCMC) without the use of approximations (linearization) was used for the estimation of the likelihood. For evaluation of the goodness-of-fit, the following plots were assessed: observed versus individual predicted concentrations, weighted residuals versus time and weighted residuals versus predictions. Individual parameter values were obtained as empirical Bayes estimates. A visual predictive check with 1000 simulated data sets was performed.

## Supplementary Information

Supplementary Information.

## Data Availability

All data generated or analyzed during this study are included in this published article (and its Supplementary Information file).
