# Supplementary material for: Development of water-soluble prodrugs of the bisdioxopiperazine topoisomerase IIβ inhibitor ICRF-193 as potential cardioprotective agents against anthracycline cardiotoxicity
Source: Sci Rep. 2021 Feb 24;11:4456. doi: 10.1038/s41598-021-83688-x (PMC7904827; doi:10.1038/s41598-021-83688-x)
Supplement: Supplementary file 1 — Supplementary Information. [file 41598_2021_83688_MOESM1_ESM.pdf]

Supplementary Material:

Development of water-soluble prodrugs of the bisdioxopiperazine topoisomerase IIβ inhibitor ICRF 193 as potential cardioprotective agents against anthracycline cardiotoxicity

Hana Bavlovič Piskáčková[1], Hana Jansová[1], Jan Kubeš[1], Galina Karabanovich[1], Nela Váňová[1], Petra Kollárová-Brázdová[2], Iuliia Melnikova[1], Anna Jirkovská[1], Olga Lenčová[2], Jaroslav Chládek[2], Jaroslav Roh[1], Tomáš Šimůnek[1], Martin Štěrba[2], Petra Štěrbová-Kovaříková[1*]

[1] Faculty of Pharmacy in Hradec Králové, Charles University, Akademika Heyrovského 1203, 500 05 Hradec Králové, Czech Republic
[2] Faculty of Medicine in Hradec Králové, Charles University, Šimkova 870/13, 500 03 Hradec Králové, Czech Republic

1 Material and Methods:

1.1 Chemicals and Materials:

Methanol, acetonitrile, formic acid (98-100%), ammonium formate, (all LC-MS grade); DMSO and EDTA dipotassium salt (HPLC grade), other chemicals used for synthesis, Millex-GV filters (PVDF, 0.22 μm), phosphate buffered saline (PBS) tablets and 4-(2-hydroxyethyl)-1-piperazineethanesulfonic acid (HEPES) were purchased from Merck (Germany). Milli-Q water was produced by a Millipore purification system (Merck- Millipore, Germany).

Blank rabbit plasma (with heparin as an anti-coagulant) was obtained from commercial source (ITEST plus, Czech Republic). Dulbecco's Modified Eagle's Medium containing Ham's F-12 nutrient mixture (DMEM/F12), fetal bovine serum (FBS), horse serum (HS), sodium pyruvate solution (100 mM; PYR) and penicillin/streptomycin solution (5000 U/mL; P/S) were purchased from Lonza (Switzerland). The sera were heat-inactivated prior to use (56 °C, 30 min). Saline buffer (further named as the buffer) was prepared in house by mixing Milli-Q water with 116 mM NaCl, 5.3 mM KCl, 1.2 mM $MgSO_4$, 1 mM $CaCl_2$, 1.13 mM NaH_2PO_4, 5mM glucose, and 20 mM HEPES (pH=7.4).

1.2 Preparation and characterization of the reaction intermediate products of the prodrugs synthesis

1.2.1 Synthesis of *meso*-4,4'-(butane-2,3-diyl)bis(1-(hydroxymethyl)piperazine-2,6-dione)

A suspension of ICRF-193 (1 g, 3.54 mmol) in DMF (5 mL) was heated to 130 °C for 10 min. Then, aqueous formaldehyde (37 %, 1.48 g, 1.43 mL, 18.23 mmol) was added to the suspension and the reaction mixture was heated to 130 °C for 4 h. The volatiles were evaporated under reduced pressure and EtOH (20 mL) was added. The mixture was kept at 4 °C overnight, the precipitated product was filtered, washed with EtOH (5 mL) and Et_2O (10 mL), and dried on air.

Yield: 67 % (0.26 g) as a white-colored solid; mp 319-321 °C. ^{1}H NMR (500 MHz, DMSO-d_6) δ 6.17 (t, J = 7.3 Hz, 2H), 5.01 (d, J = 6.3 Hz, 4H), 3.47 (dd, J = 16.4, 1.7 Hz, 4H), 3.36 (dd, J = 16.6, 1.8 Hz, 4H), 2.80 - 2.74 (m, 2H), 0.89 (d, J = 5.7 Hz, 6H). ^{13}C NMR (126 MHz, DMSO-d_6) δ 170.90, 61.22, 58.79, 52.53, 9.48.

1.2.2 General Method for the Synthesis of Boc-Protected GK-667, GK-678 and GK-691

A solution of dicyclohexyl carbodiimide (0.72 g, 3.45 mmol) in CH_2Cl_2 (30 mL) was added to the suspension of *meso*-4,4'-(butane-2,3-diyl)bis(1-(hydroxymethyl)piperazine-2,6-dione) (0.6 g, 1.75 mmol), Boc-protected amino acid (3.54 mmol) and 4-dimethylaminopyridine (6 mg, 0.049 mmol) in CH_2Cl_2 (50 mL) at 0 °C and under argon atmosphere. The reaction mixture was stirred at rt for 48 h. The reaction mixture was filtered, and the filtrate was washed with a mixture H_2O/CH_3COOH, 15:1 (48 mL). The organic layer was separated, dried over anhydrous Na_2SO_4 and concentrated under reduced pressure. The product was purified using column chromatography.

Boc-Protected GK-667 (*meso*-2,3-bis(4-(2-(*tert*-butoxycarbonylamino)acetoxymethyl)-3,5-dioxopiperazin-1-yl)butane). The product was purified using column chromatography (mobile phase: $CHCl_3$/MeOH, 70:1). Yield: 54 % as a white solid. ^{1}H NMR (500 MHz, DMSO-d_6) δ 7.21 (t, J = 6.2 Hz, 2H), 5.68 – 5.58 (m, 4H), 3.65 (d, J = 6.2 Hz, 4H), 3.58 – 3.51 (m, 4H), 3.48 – 3.41 (m, 4H), 2.84 – 2.76 (m, 2H), 1.37 (s, 18H), 0.90 (d, J = 5.1 Hz, 6H). ^{13}C NMR (126 MHz, DMSO-d_6) δ 170.52, 169.66, 155.94, 78.49, 62.20, 58.84, 52.29, 41.79, 28.31, 9.70.

Boc-Protected GK-678 (*meso*-2,3-bis(4-((*S*)-2-(*tert*-butoxycarbonylamino)propionyloxymethyl)-3,5-dioxopiperazin-1-yl)butane). The product was purified using column chromatography (mobile phase: $CHCl_3$/MeOH, 30:1). Yield: 65 % as a white solid. ^{1}H NMR (500 MHz, DMSO-d_6) δ 7.29 (d, J = 7.4 Hz, 2H), 5.73 – 5.60 (m, 2H), 5.56 (dd, J = 9.5, 4.5 Hz, 2H), 3.95 (t, J = 7.3 Hz, 2H), 3.55 (d, J = 16.7 Hz, 4H), 3.45 (d, J = 16.8 Hz, 4H), 2.84 – 2.76 (m, 2H), 1.36 (s, 18H), 1.17 (d, J = 7.3 Hz, 6H), 0.90 (d, J = 4.7 Hz, 6H). ^{13}C NMR (126 MHz, DMSO-d_6) δ 172.41, 170.49, 155.34, 78.38, 62.24, 58.85, 52.28, 49.01, 28.32, 16.75, 9.71.

Boc-Protected GK-691 (*meso*-2,3-bis(4-((*S*)-2-(*tert*-butoxycarbonylamino)-3-phenylpropionyloxymethyl)-3,5-dioxopiperazin-1-yl)butane). The product was purified using column chromatography (mobile phase: $CHCl_3$/MeOH, 100:1). Yield: 30 % as a white solid. ^{1}H NMR (500 MHz, $CDCl_3$) δ 7.34 – 7.20 (m, 6H), 7.20 – 7.12 (m, 4H), 5.82 (t, J = 3.2 Hz, 4H), 4.95 (d, J = 8.4 Hz, 2H), 4.60 (q, J = 6.8 Hz, 2H), 3.58 – 3.49 (m, 4H), 3.47 – 3.39 (m, 4H), 3.16 – 2.95 (m, 4H), 2.67 – 2.59 (m, 2H), 1.41 (s, 18H), 1.07 (d, J = 2.1 Hz, 6H). ^{13}C NMR (126 MHz, $CDCl_3$) δ 170.72, 169.07, 154.90, 135.59, 129.39, 128.55, 127.00, 79.95, 62.58, 60.38, 53.98, 53.09, 38.08, 28.26, 9.68.

Purity of the synthetized prodrugs were tested by: 1/ direct infusion of the prodrugs into MS (Figure S8); 2/ NMR scan of the prodrugs (Figure S9) and 3/ UHPLC-MS analysis of the prodrugs to assay the presence of ICRF-193 (Figure S10).

Although ESI-MS spectra showed in addition to $[M+H]^+$ and corresponding adducts also other ions of lower *m/z*, these are the products of in source fragmentation. This was confirmed by H and C NMR scans (Figure S9) as well as UHPLC-MS assay. The last test evidenced that the synthesized prodrugs contain less than 0.5% of the active ingredient ICRF-193 (Figure S10).

1.3 Preparation and characterization of the internal standard I.S.$_{(A)}$ and the metabolite ICRF-193$_{met}$

1.3.1 Synthesis of I.S.$_{(A)}$ (*meso-N,N'*-bis(methylcarbamoylmethyl)-2,3-diaminobutane-*N,N'*-diacetic acid)

Meso-4,4'-(butane-2,3-diyl)bis(1-methylpiperazine-2,6-dione).
Diisopropyl azodicarboxylate (0.52 g, 0.5 mL, 2.57 mmol) was added dropwise to the mixture of ICRF-193 (0.2 g, 0.71 mmol), triphenylphosphine (0.67 g, 2.56 mmol) and methanol (0.2 g, 0.25 mL, 6.24 mmol) in THF (30 mL) under inert atmosphere. The reaction mixture was stirred at rt for 24 h, then filtered and the filtrate was concentrated under reduced pressure. The residue was dissolved in EtOAc (50 mL) and washed with 2% HCl (1 × 30 mL) and water (2 × 20 mL). Organic layer was separated, dried over anhydrous Na_2SO_4 and concentrated under reduced pressure. The product was purified using column chromatography (hexane/EtOAc, 2:1). Yield: 13 % (0.028 g) as a white solid; mp 255-258 °C. ^1H NMR (500 MHz, DMSO-d_6) δ 3.46 (dd, J = 16.4, 1.8 Hz, 4H), 3.36 (dd, J = 16.4, 1.8 Hz, 4H), 2.97 (s, 6H), 2.78 – 2.73 (m, 2H), 0.88 (d, J = 6.0 Hz, 6H). ^{13}C NMR (126 MHz, DMSO-d_6) δ 171.35, 58.72, 52.35, 25.29, 9.42.

I.S.$_{(A)}$ *(meso-N,N'*-bis(methylcarbamoylmethyl)-2,3-diaminobutane-*N,N'*-diacetic acid). *Meso*-4,4'-(butane-2,3-diyl)bis(1-methylpiperazine-2,6-dione) (0.057 g, 0.184 mmol) and 1M NaOH (0.4 mL) in H_2O (2 mL) was stirred at rt overnight. The reaction mixture was acidified with Amberlyst 15 (hydrogen form) to pH 4. Amberlyst 15 was filtered off and the clear aqueous filtrate was evaporated to dryness under reduced pressure. The product was dried under reduced pressure over P_2O_5.

1.3.2 Synthesis of ICRF-193$_{met}$ (*meso-N,N'*-bis(carbamoylmethyl)-2,3-diaminobutane-*N,N'*-diacetic acid)

The mixture of *meso*-4,4'-(butane-2,3-diyl)bis(piperazine-2,6-dione) (0.2 g, 0.71 mmol) and 1M NaOH (1.8 mL, 2.5 eq.) in H_2O (4 mL) was stirred at rt for 24 h. The reaction mixture was acidified with Amberlyst 15 (hydrogen form) to pH 4. Amberlyst 15 was filtered off and the clear aqueous filtrate was evaporated to dryness under reduced pressure. Product was further dried under reduced pressure over P_2O_5. Yield: 44 % (0.1 g). ^{1}H NMR (500 MHz, D_2O) δ 3.72 – 3.64 (m, 4H), 3.64 – 3.52 (m, 4H), 3.20 (q, *J* = 6.5 Hz, 2H), 1.28 (d, *J* = 5.5 Hz, 6H). ^{13}C NMR (126 MHz, D_2O) δ 173.15, 172.66, 62.00, 54.80, 54.41, 10.94.

1.4 UHPLC-MS/MS methods and validation

1.4.1 Preparation of stock and working solutions

Stock solutions (3 mM) were prepared by dissolving of relevant amount of the substance in following solvents: methanol (I.S.$_{(A)}$), methanol/water mixture (1:1, v/v) (ICRF-193$_{met}$ and I.S.$_{(B)}$), DMSO (ICRF-193) and water (all prodrugs). The stock solutions were stable up to one month when stored at –20 °C; except for the stock solutions of the prodrugs that were freshly prepared before each experiment. Working solutions of concentration range of 0.25 – 2500 µM and 20-450 µM of the analytes were prepared by appropriate dilution of the stock solutions with the same solvent. All working solutions, except for the prodrugs, were stable up to 7 days when stored at 4 °C.

1.4.2 Method development

The assay development was complicated by opposite chromatographic behavior of the analytes. While highly polar ICRF-193$_{met}$ was poorly retained, more lipophilic active compound and mainly its prodrugs had stronger affinity to the column. The following chromatographic columns with capability of increasing the retention of polar analytes were tested: Luna Omega Polar (100 × 2.1 mm, 1.6 µm), Kinetex F5 column (100 mm × 2.1 mm, 1.7 µm) both from Phenomenex (USA) and Zorbax SB Aq (3× 100 mm, 1.8 µm) from Agilent (USA). Mobile phases composed of mixtures of either water, formic acid (0.01-1%) or ammonium formate (0.5-10 mM) with methanol or acetonitrile were examined on these columns in different gradient profiles. Zorbax SB Aq previously used for analysis of sobuzoxane[1] failed to provide appropriate peak shapes of the analytes. Kinetex F5 allowed acceptable retention of both ICRF-193 and ICRF-193$_{met}$ but was inappropriate for the prodrugs due to their strong retention

and peak tailing. The best peak shapes together with acceptable retention of all analytes was achieved with Luna Omega Polar column. Another analytical issue was low sensitivity of detection and deteriorated peak shape of the metabolite ICRF-193$_{met}$. This was caused by complexation of trace metal ions within a chromatographic system by the metabolite. However, this was resolved by flushing the column with a mixture of 2 mM di-potassium EDTA solution and acetonitrile (80:20; v/v) prior to the first analysis (4 h using flow rate of 0.25 mL/min) [2]. Moreover, different solvent for sample preparation was also needed for each analyte. While sample acidification improved prodrugs peak shapes and their post-preparative stability, it suppressed signal of detection of ICRF-193$_{met}$. Therefore, acidification with 0.5% formic acid was found as a compromise for simultaneous assay of the prodrugs with ICRF-193$_{met.}$ Furthermore, higher sensitivity of detection of ICRF-193 and ICRF-193$_{met}$ was reached with methanol but tremendous peak tailing was observed for all prodrugs. Therefore, to achieve good peak shape of the prodrugs, acetonitrile was chosen as a mobile phase for the method I, while the methanol was used for the method II.

1.4.3 Method validation

For initial comparison of ICRF-193 release from all prodrugs *in vitro*, the method I was partially validated for simultaneous assay of GK-667, GK-678 and GK-691, together with ICRF-193 and the metabolite ICRF-193$_{met}$ in DMEM. The selectivity of the method was confirmed due to insignificant coelution in the blank matrices at the retention times of the analytes and internal standard (I.S.). Linearity was verified within the concentration range of 0.5 to 100 μM for all analytes in DMEM. Linear fits using weighted standard curves ($1/x^2$) with corresponding R^2>0.99 were obtained, and the back-calculated concentrations were within ± 15 % of the nominal value, which is in line with the recommendation of the European Medicines Agency (EMA) for bioanalytical method validation[3].

Subsequently, the method I was fully validated for assay of the chosen prodrug GK-667 the active form ICRF-193 and its metabolite ICRF-193$_{met}$ in DMEM, the buffer, rabbit plasma and NVCMs and the method II only for ICRF-193 and ICRF-193$_{met}$ in rabbit plasma. Following concentration ranges were used: 0.2 – 100 μM for all compounds in DMEM, the buffer and plasma, 10-100 pmol/10^6cells for all compounds in NVCMs (method I) and 0.01-15 μM and 0.01-1 μM for ICRF-193 and ICRF-193$_{met}$ in plasma, respectively (method II). Selectivity of the methods was explored by examination of the detector response from analysis of blank matrices at the retention times of the analytes.

Linearity was tested within the concentration range of 0.2 – 100 μM for all compounds in DMEM, the buffer and plasma and 10-100 pmol/10^6cells in NVCMs (method I). For method II, the concentration ranges 0.01-15 μM and 0.01-1 μM were used for determination of ICRF-193 and ICRF-193_{met} in plasma, respectively. Unweighted and weighted (1/x and $1/x^2$) linear regression was applied to obtain the best fitting calibration model.

Accuracy and precision were calculated at 4 levels (LLOQ, low, medium and high concentrations). Five quality control (QC) samples were used for all concentration levels. Only four QC samples were used for NVCMs due to the limited availability of the samples. Accuracy was expressed as the mean percentage of the determined amount relative to spiked amount of the compound and precision as the relative standard deviation (RSD).

The matrix effect was investigated for six different lots of DMEM and buffer, plasma samples taken from six different animals and cardiac cells from five different isolations. The matrix effect was calculated from the ratio of the peak area of spiked blank sample after extraction procedure and the peak area of the neat standard solution: it was expressed as an RSD among I.S.-normalized matrix effects.

Recovery from plasma and NVCMs (at either low or medium concentration) was calculated as the mean percentage of ratio of the peak area of the spiked blank sample before and after extraction procedure.

Stability of the analytes was examined at low and high concentrations. Process stability (n=3) in plasma was tested for 20 min at either laboratory temperature (23 °C) or on ice (4 °C, only for GK-667). Post-preparation stability was examined for 8 to 10 h in an autosampler (8 °C, n=3) for all treated matrices. Freeze-thaw stability of ICRF-193 and ICRF-193_{met} in plasma was tested at -80 °C and the freezing cycle was repeated twice (n=3). The buffer, DMEM, NVCMs and plasma samples containing GK-667 from *in vitro* studies and from first intervals of pharmacokinetic study (up to 20 min) were treated and analyzed immediately after collection, thus the freeze-thaw stability did not need to be examined.

1.5 LDH activity evaluation

The control wells were treated with lysis buffer (0.1 M potassium phosphate, 1% Triton X-100, 1 mM DTT, 2 mM EDTA; pH 7.8; 15 min; room temperature) to measure the total cellular LDH level (sum of the activity in the control medium and the lysate). The activity of LDH was assayed in Tris-HCl buffer (pH 8.9) containing 35 mM sodium lactate and 5 mM NAD^+. The rate of NAD^+ reduction was monitored spectrophotometrically at 340 nm for 2 min. The slope of the linear region and molar absorption coefficient e = $6.22*10^3$ M/cm were used to calculate the LDH activity, and the data were expressed as a percentage of total LDH activity.

References:

1 Reimerova, P. *et al.* UHPLC-MS/MS method for analysis of sobuzoxane, its active form ICRF-154 and metabolite EDTA-diamide and its application to bioactivation study. *Sci Rep* **9**, 4524, doi:10.1038/s41598-019-40928-5 (2019).

2 Kovarikova, P. *et al.* Development of LC-MS/MS method for the simultaneous analysis of the cardioprotective drug dexrazoxane and its metabolite ADR-925 in isolated cardiomyocytes and cell culture medium. *J Pharm Biomed Anal* **76**, 243-251, doi:10.1016/j.jpba.2012.12.024 (2013).

3 European Medicines Agency. Guideline on bioanalytical method validation. http://www.ema.europa.eu/docs/en_GB/document_library/Scientific_guideline/2011/08/WC500109686.pdf (2012)

Figures

Figure S1: Representative chromatograms of UHPLC-MS/MS analyses of the prodrugs

Chromatograms of UHPLC-MS/MS analysis of the prodrugs, ICRF-193, ICRF-193$_{met}$ and the internal standards (concentration of 25 µM) in the cell medium (DMEM). **(a)** GK-667, **(b)** GK-678 and **(c)** GK-691.

(1) I.S.$_{(A)}$, *(2)* ICRF-193$_{met}$, *(3)* ICRF-193, *(4)* I.S.$_{(B)}$, *(5a)* GK-667, *(5b)* GK-678, *(5c)* GK-691.

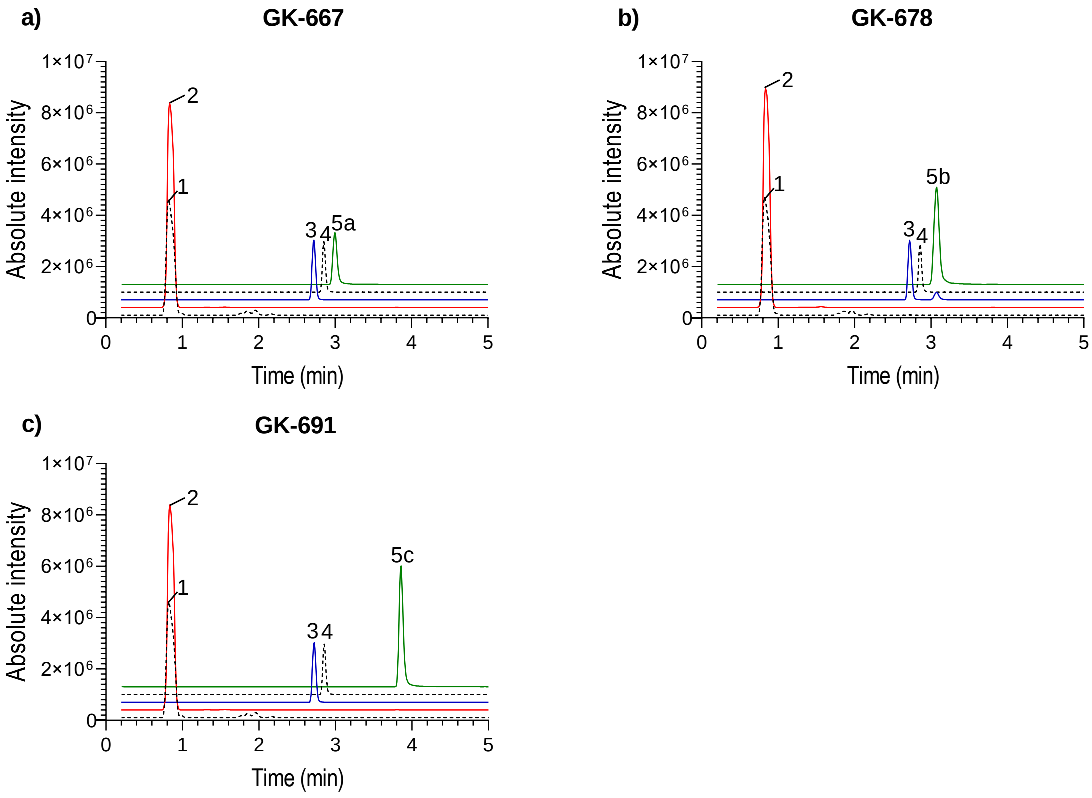

Figure S2: Toxicities of the prodrugs in neonatal ventricular rat cardiomyocytes (NVCMs).

Toxicities of prodrugs **(a)** GK-667, **(b)** GK-678, **(c)** GK-691 in NVCMs. The cytotoxicity was determined by measuring of the lactate dehydrogenase (LDH) release into the culture medium. Data are presented as the mean +/- SD of four independent experiments. Statistical significance was evaluated using one-way ANOVA and Holm-Sidak's post-hoc test; c - compared to control; P ≤ 0.01.

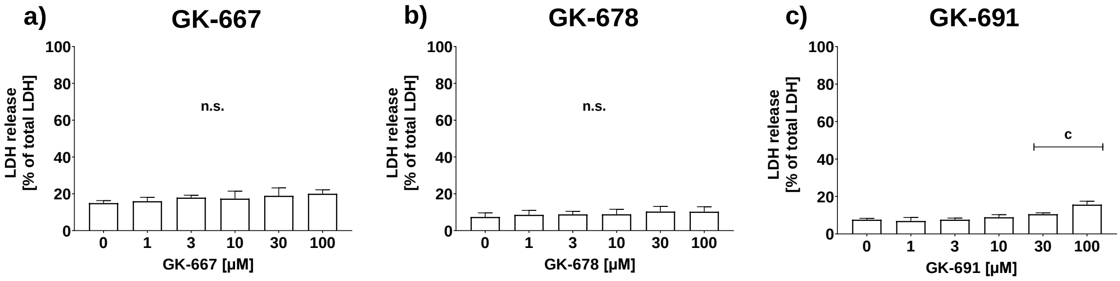

Figure S3: UHPLC-MS/MS analysis of GK-667, ICRF-193 and ICRF-193$_{met}$ in different biological materials.

Representative chromatograms of UHPLC-MS/MS analysis of the compounds and the corresponding blanks in **(a, b)** the cell medium – DMEM, **(c, d)** the buffer, **(e, f)** neonatal ventricular cardiomyocytes (NVCMs) and rabbit plasma using either **(g, h)** method I or **(i, j)** method II. The chromatograms show analysis at the concentrations of 10 μM (DMEM, the buffer and rabbit plasma-method I), 20 pmol/10^6 cells (NVCMs) and 0.5 μM (rabbit plasma-method II) of the analytes.

(1) I.S.$_{(A)}$, *(2)* ICRF-193$_{met}$, *(3)* ICRF-193, *(4)* I.S.$_{(B)}$, *(5)* GK-667, *(1b)* blank of I.S.$_{(A)}$, *(2b)* blank of ICRF-193$_{met}$, *(3b)* blank of ICRF-193, *(4b)* blank of I.S.$_{(B)}$, *(5b)* blank of GK-667.

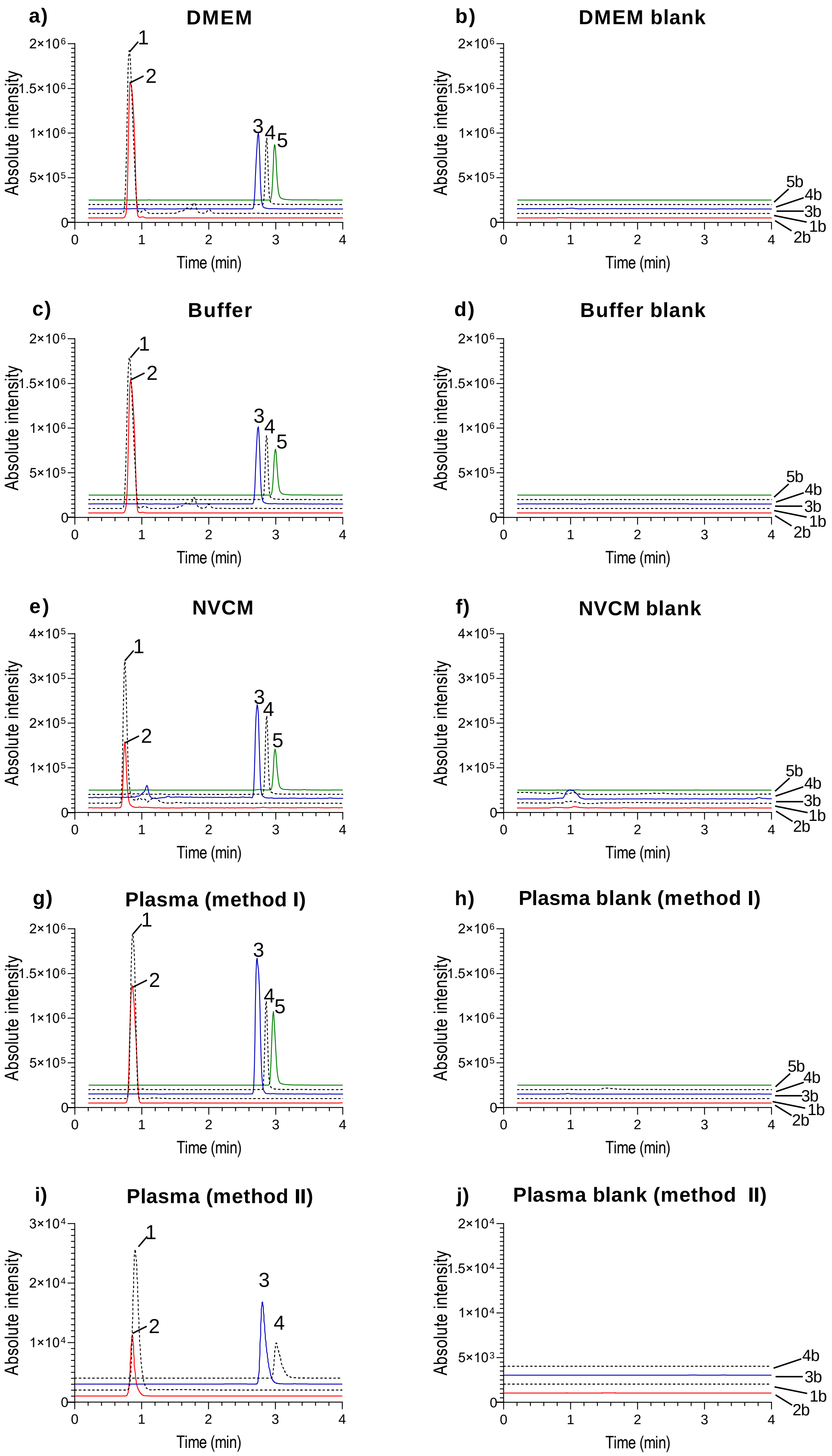
a)
DMEM
b)
DMEM blank
c)
Buffer
d)
Buffer blank
e)
NVCM
f)
NVCM blank
g)
Plasma (method I)
h)
Plasma blank (method I)
i)
Plasma (method II)
j)
Plasma blank (method II)
Absolute intensity
Time (min)
1
2
3
4
5
5b
4b
3b
1b
2b
2×10⁶
1.5×10⁶
1×10⁶
5×10⁵
4×10⁵
3×10⁵
2×10⁵
1×10⁵
3×10⁴
2×10⁴
1×10⁴
1.5×10⁴
5×10³
0
1
2
3
4

Figure S4: Process stability study.

Stability of **(a)** GK-667, ICRF-193 and ICRF-193$_{met}$ in untreated plasma at laboratory temperature (23 °C) and **(b)** of GK-667 at 4 °C for 20 min (n=3).

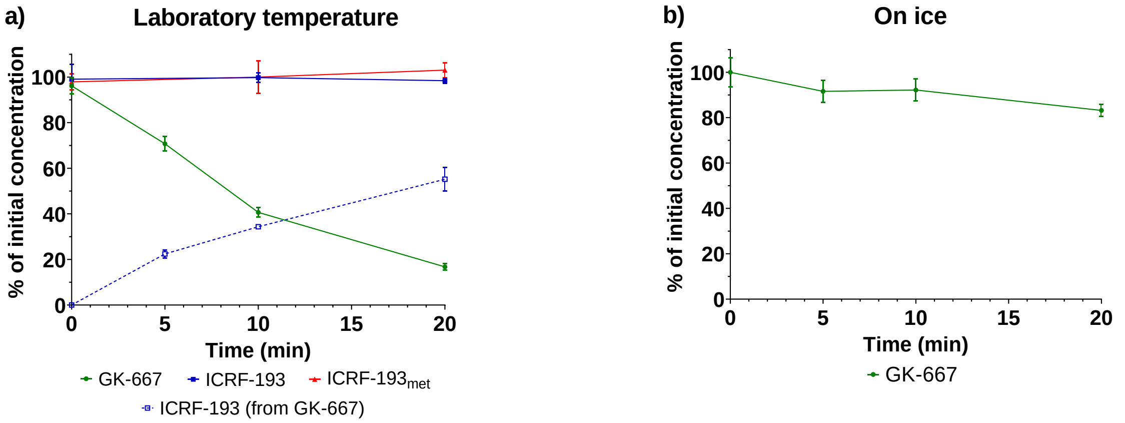

Figure S5: Schematic representation of the four-compartmental parent-metabolite model for ICRF-193 and ICRF-193$_{met}$.

Hydrolysis of GK-667 is very fast and the parent compound was essentially completely cleared from plasma within 10 min after injection. ICRF-193 was quantitatively dominating at the first sampling interval (5 min). Therefore, the model assumes instant input and complete absolute bioavailability of ICRF-193 (F=1). V_1, V_2 and V_3 are volumes of central and peripheral compartments for ICRF-193 and, V_4 is the distribution volume for ICRF-193$_{met}$. The clearance characteristics are as follows: CL_{met} describes formation of ICRF-193$_{met}$ from ICRF-193; CL_{other} summarizes clearances of ICRF-193 via other elimination pathways; Q_{12} and Q_{13} are inter-compartmental clearances; $CL_{ICRF-193met}$ is the total clearance of the metabolite.

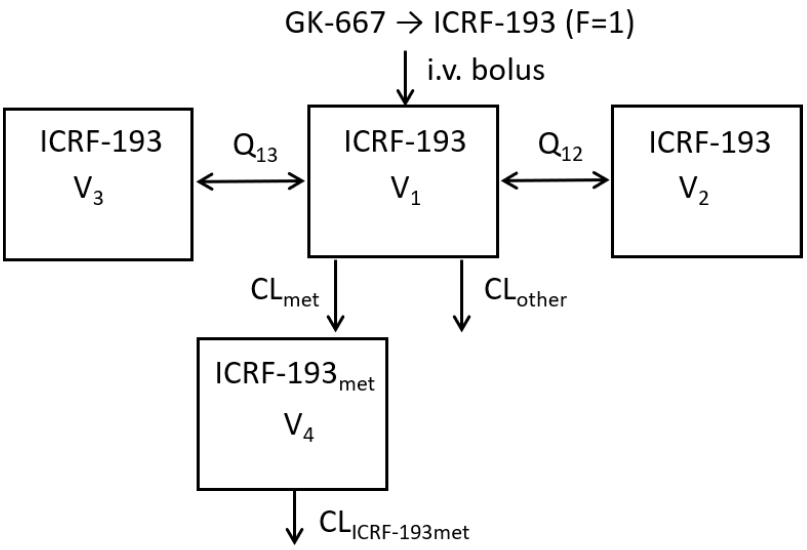

Figure S6: Visual predictive performance of the population model.

Scatter plots of the individual predicted versus observed concentrations for **(a)** ICRF-193 and **(b)** ICRF-193$_{met}$.

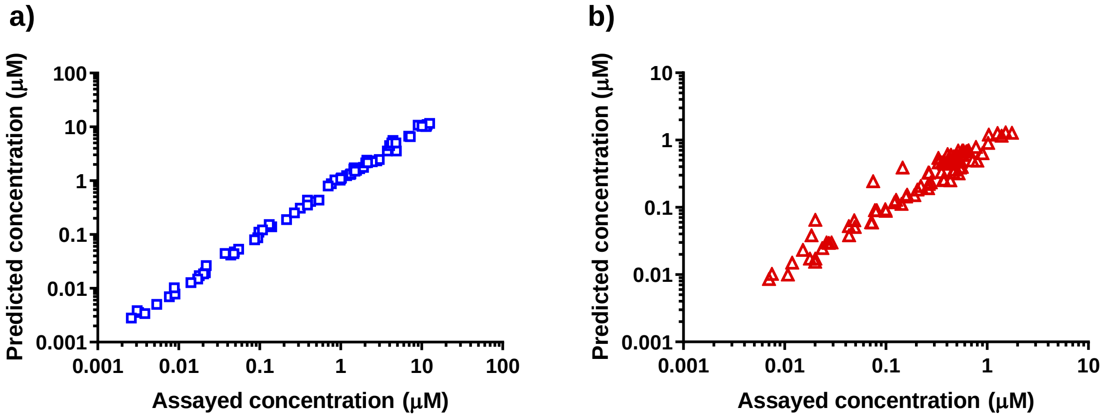

Figure S7: Chemical structures of internal standards used for UHPLC-MS/MS analysis

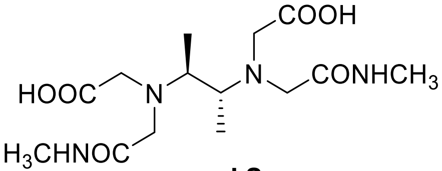

I.S.$_{(A)}$

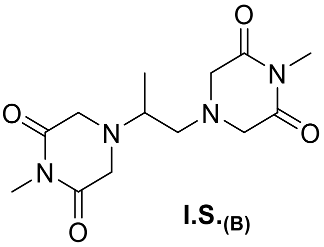

I.S.$_{(B)}$

Figure S8: ESI-MS spectra of prodrugs (a) GK-667, (b) GK-678 and (c) GK-691

The methanol solution of each prodrug (1 µg/ml) acidified with formic acid (0.5%) was directly infused to LCQ Advantage Max mass spectrometer (Thermo Finnigan, USA).

a) GK-667

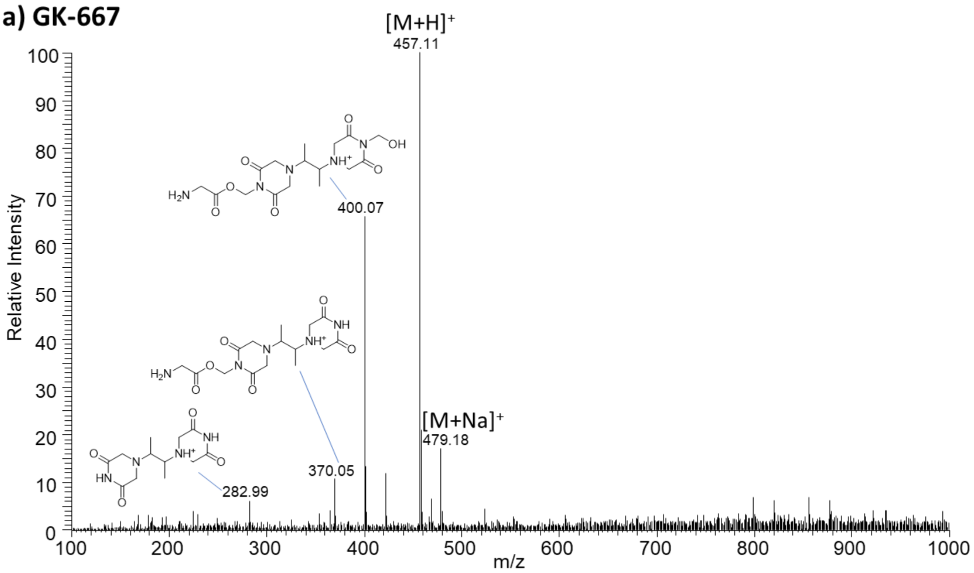

b) GK-678

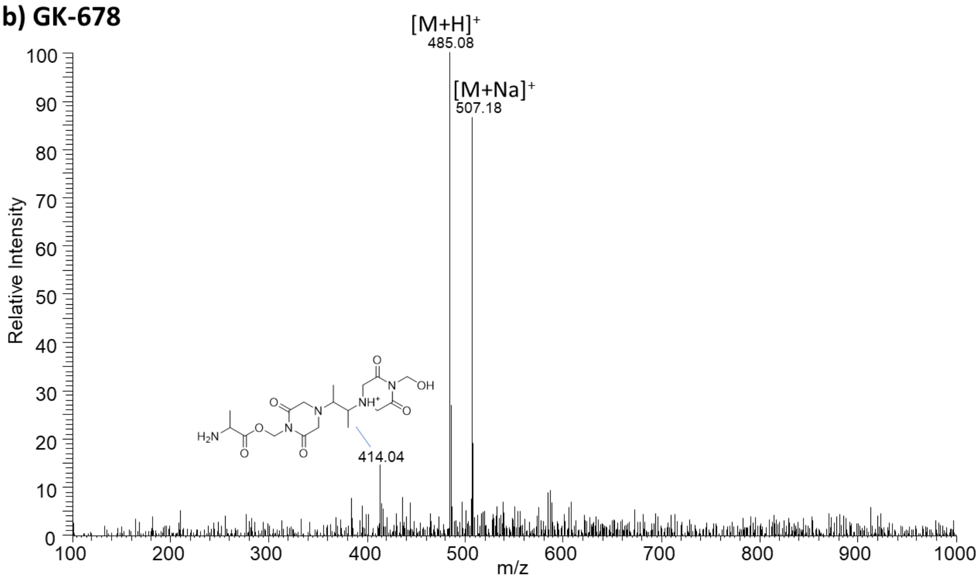

c) GK-691

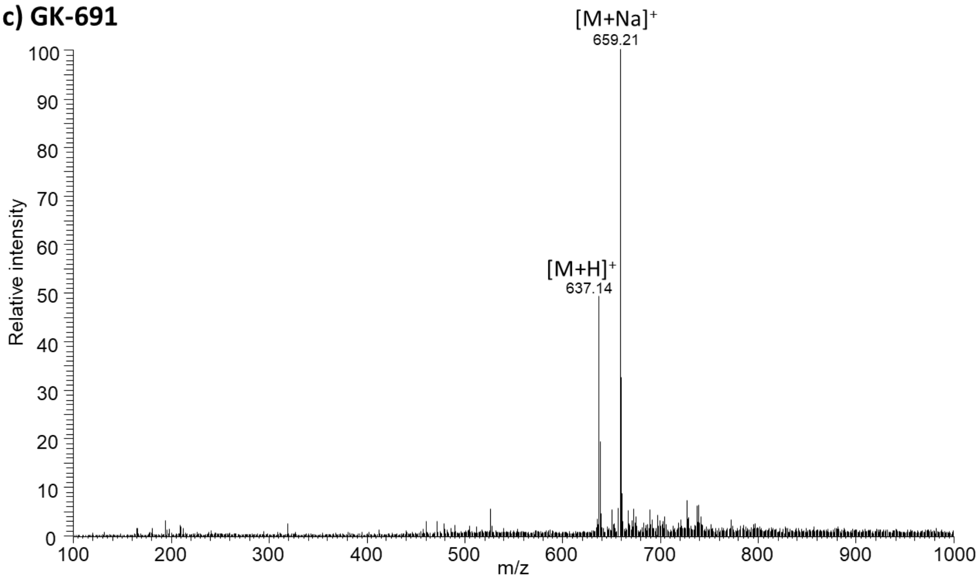

Figure S9: Investigation of prodrugs' purity by NMR.

Scans of H and C NMR of the prodrugs (a, b) GK-667, (c, d) GK-678 and (e, f) GK-691

a) GK-667: ^{1}H NMR (500 MHz, DMSO-d_6)

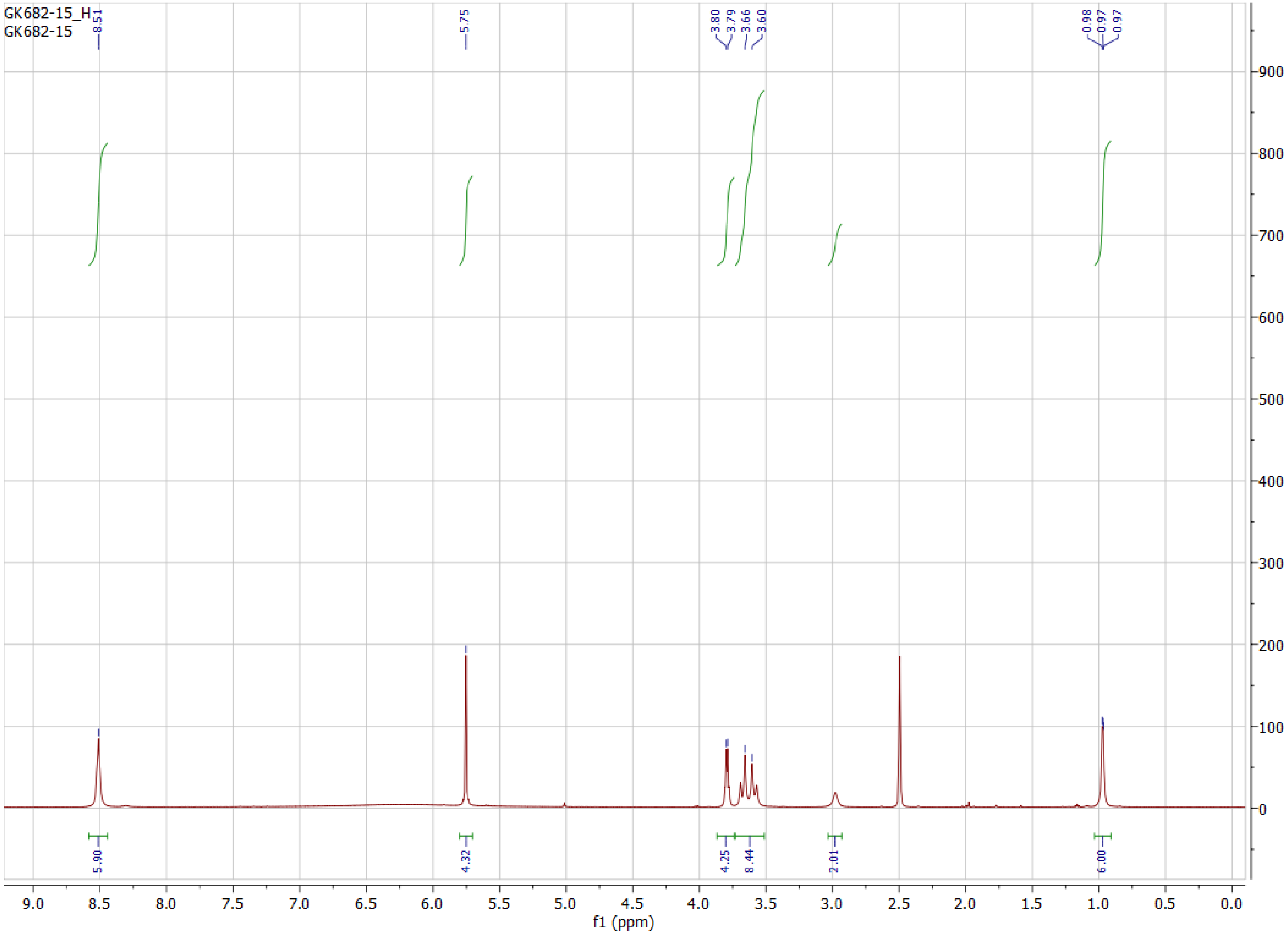

GK682-15_H
GK682-15
8.51
5.75
3.80
3.79
3.66
3.60
0.98
0.97
0.97
5.90
4.32
4.25
8.44
2.01
6.00
f1 (ppm)

b) GK-667: ^{13}C NMR (126 MHz, DMSO-d_6)

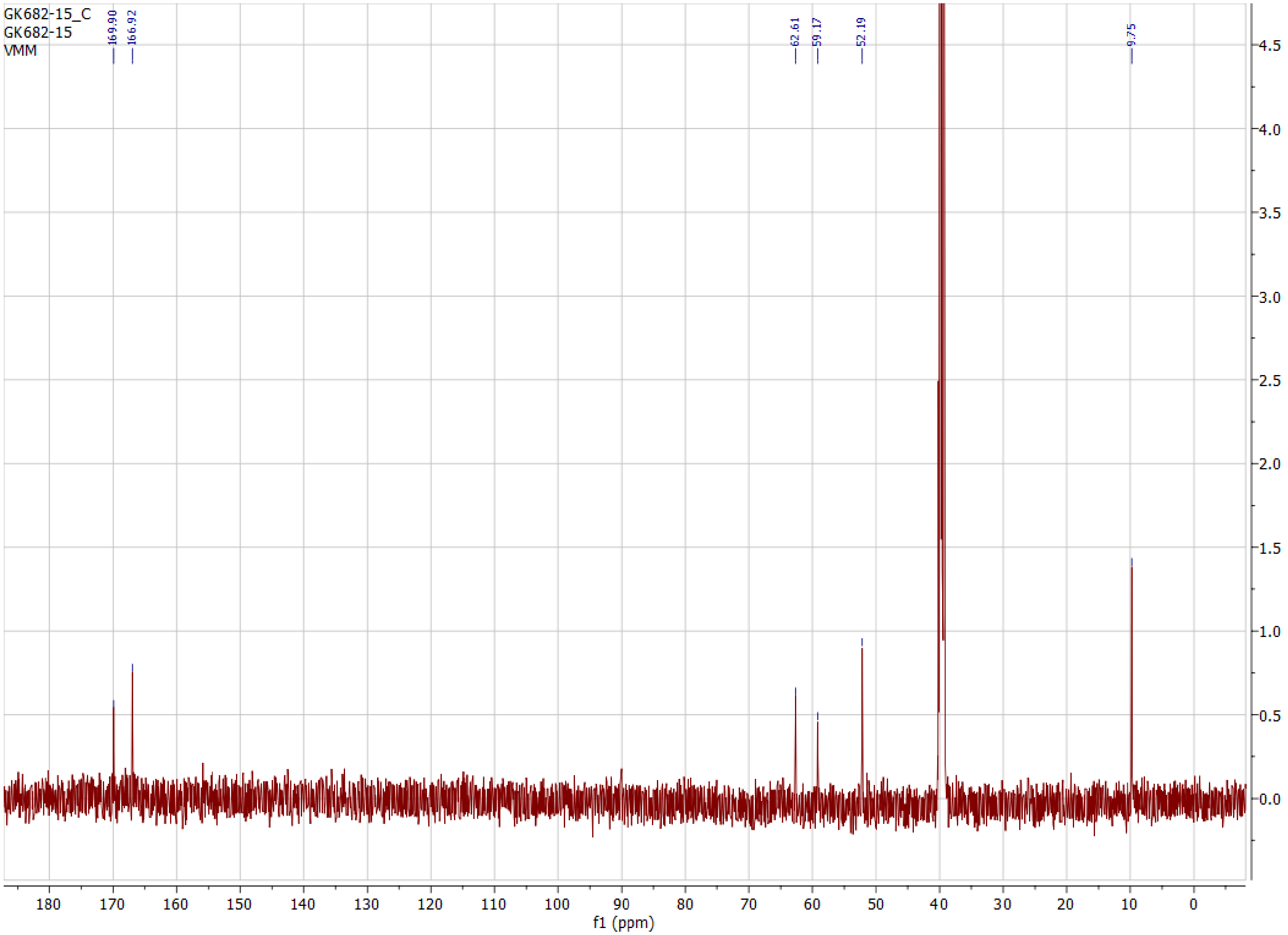

c) GK-678: ^{1}H NMR (500 MHz, DMSO-d_6)

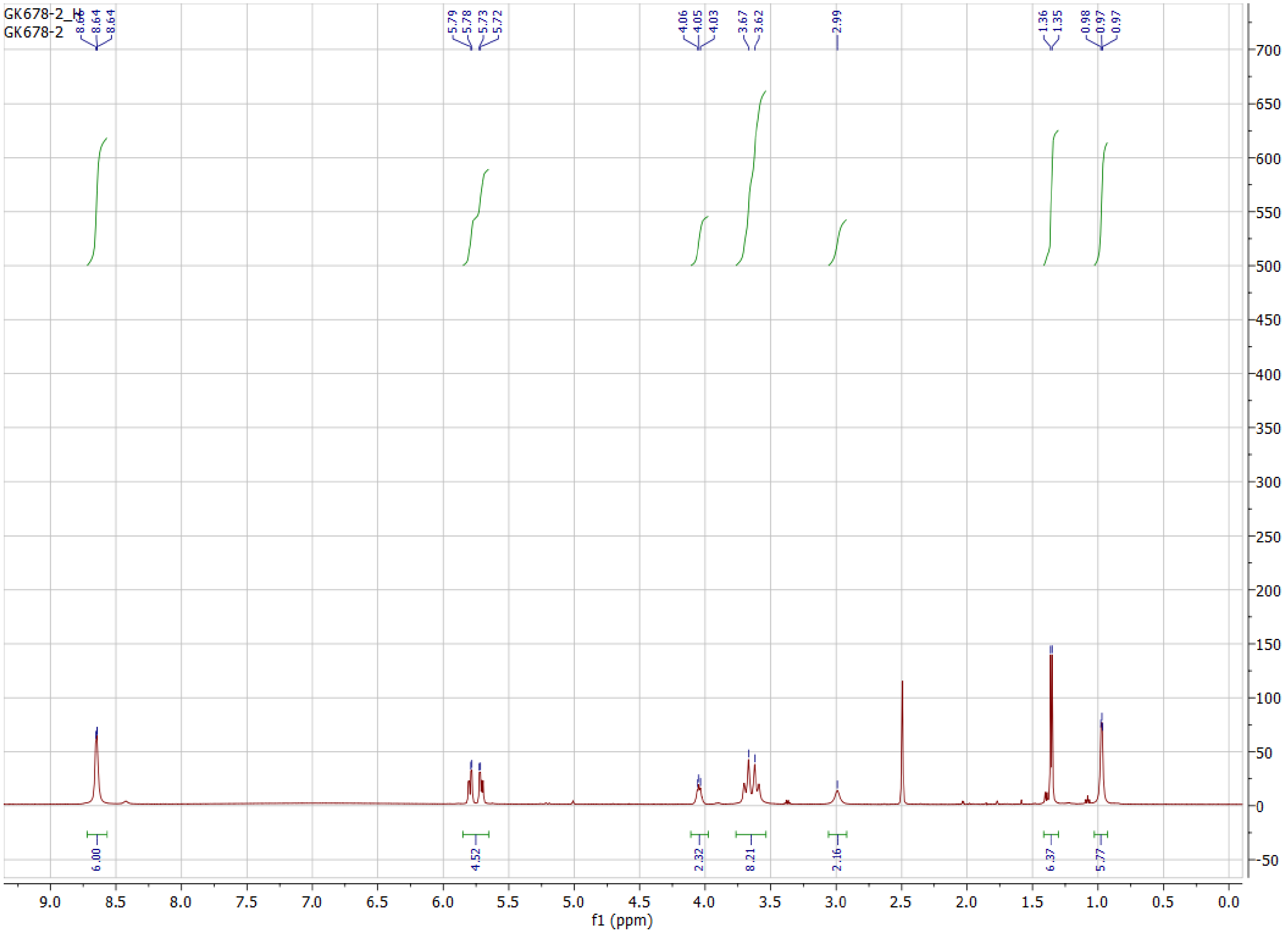

d) GK-678: ^{13}C NMR (126 MHz, DMSO-d_6)

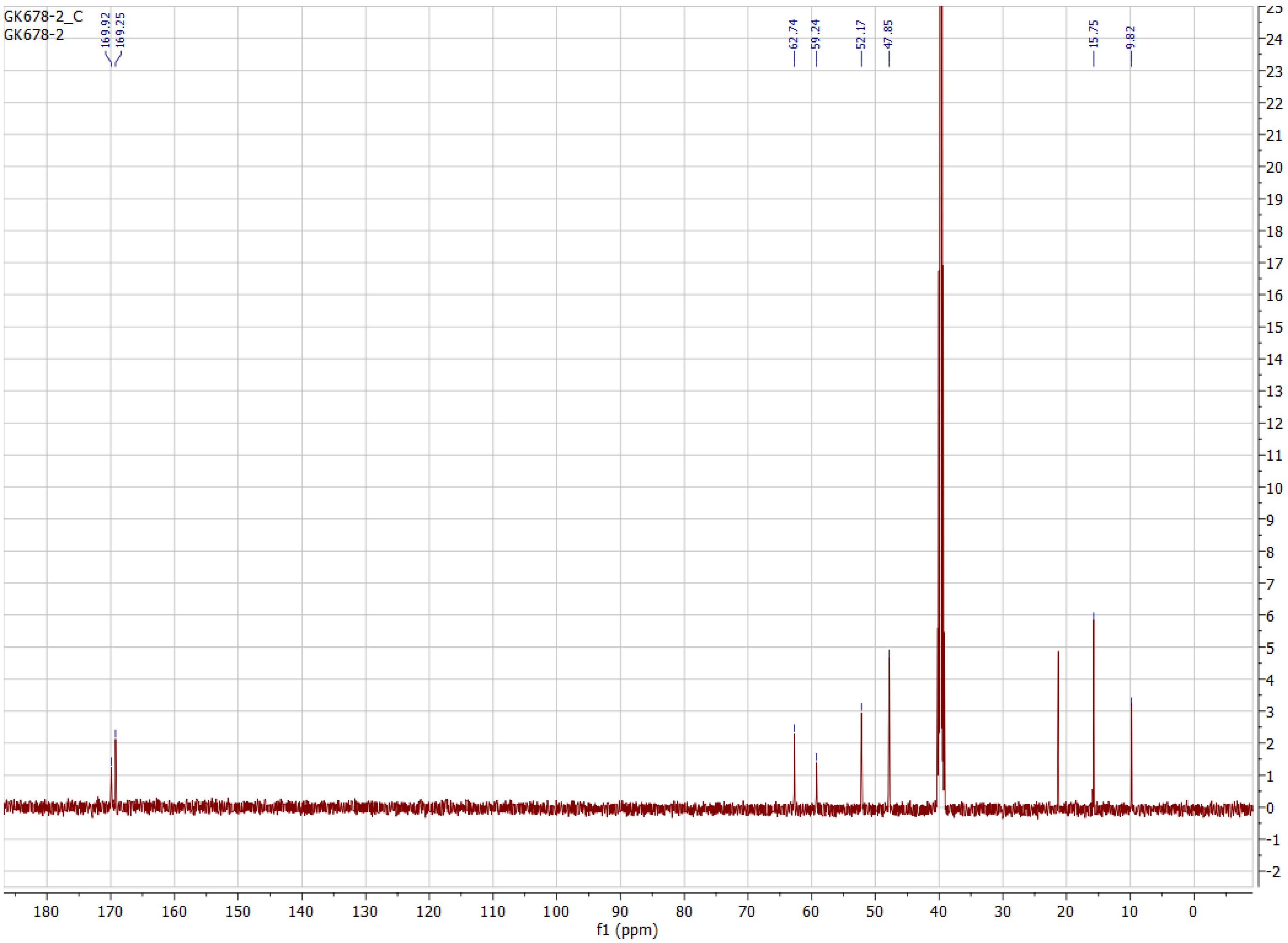

e) GK-691: ^{1}H NMR (500 MHz, DMSO-d_6)

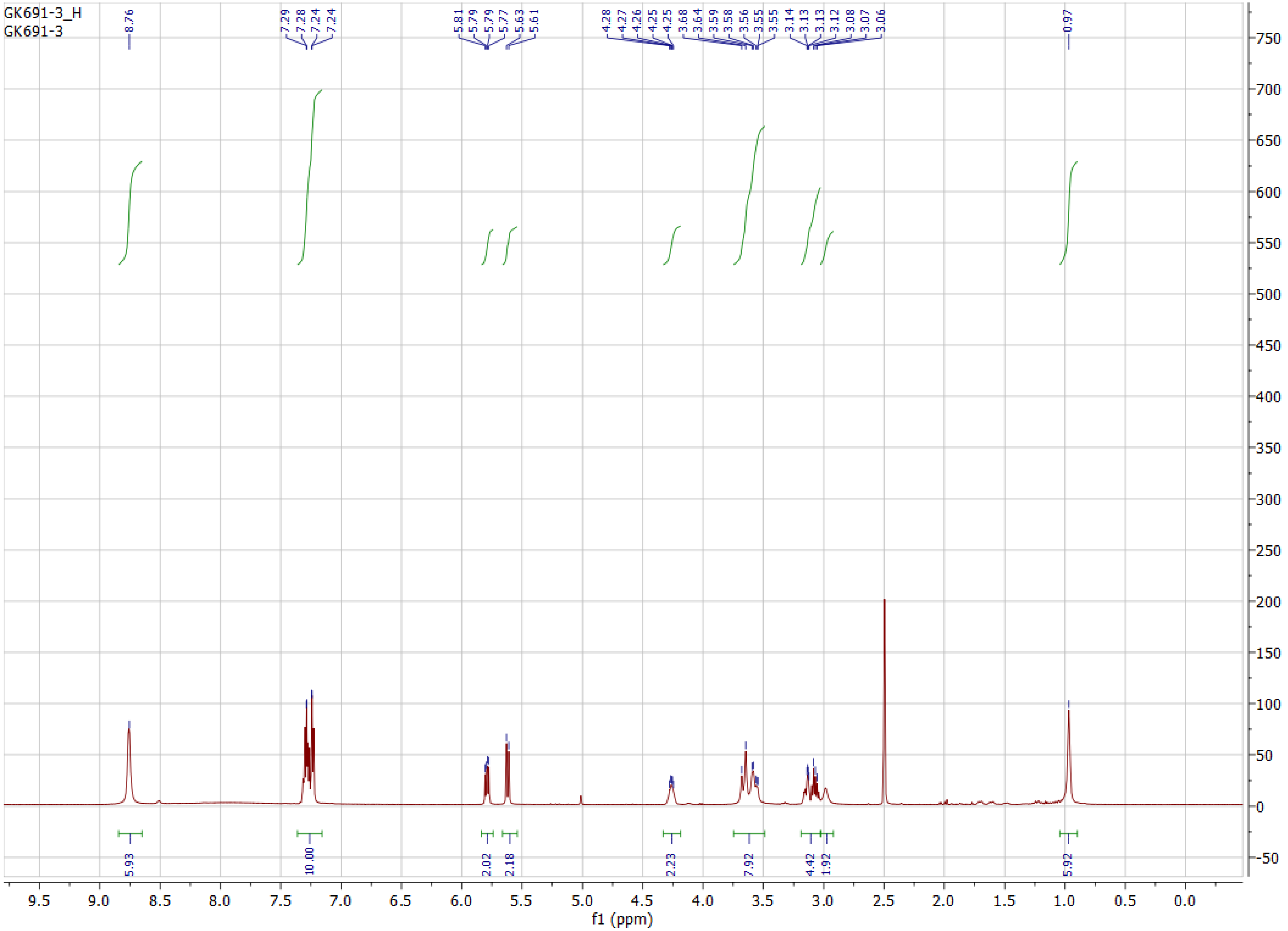

f) GK-691: ^{13}C NMR (126 MHz, DMSO-d_6)

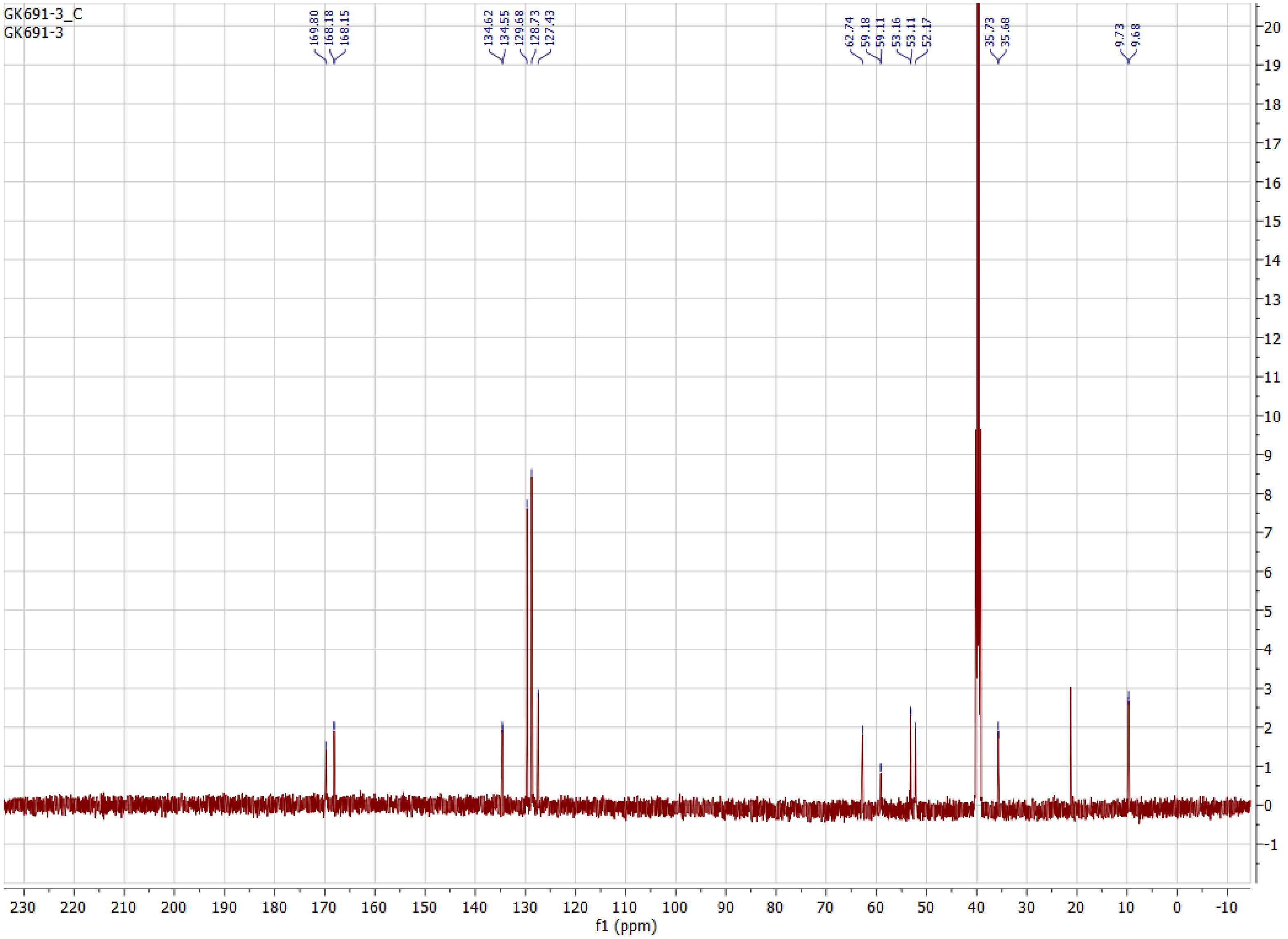

Figure S10: UHPLC-MS/MS analysis of the prodrugs' purity: - detection of the presence of ICRF-193 in: (a) GK-667, (b) GK-678 and (c) GK-691 and the analysis of the standard of ICRF-193 (0.5 µM) for comparison. The freshly prepared water solution of each prodrug (100 µM) was acidified with formic acid (0.5%) and immediately analyzed by method I. The zoomed chromatograms demonstrated that the substances of all prodrugs contained only trace amounts of ICRF-193 (< 0.5 µM). Furthermore, the signal corresponding to ICRF-193 detected at the same retention times as the prodrug confirmed in-source fragmentation.

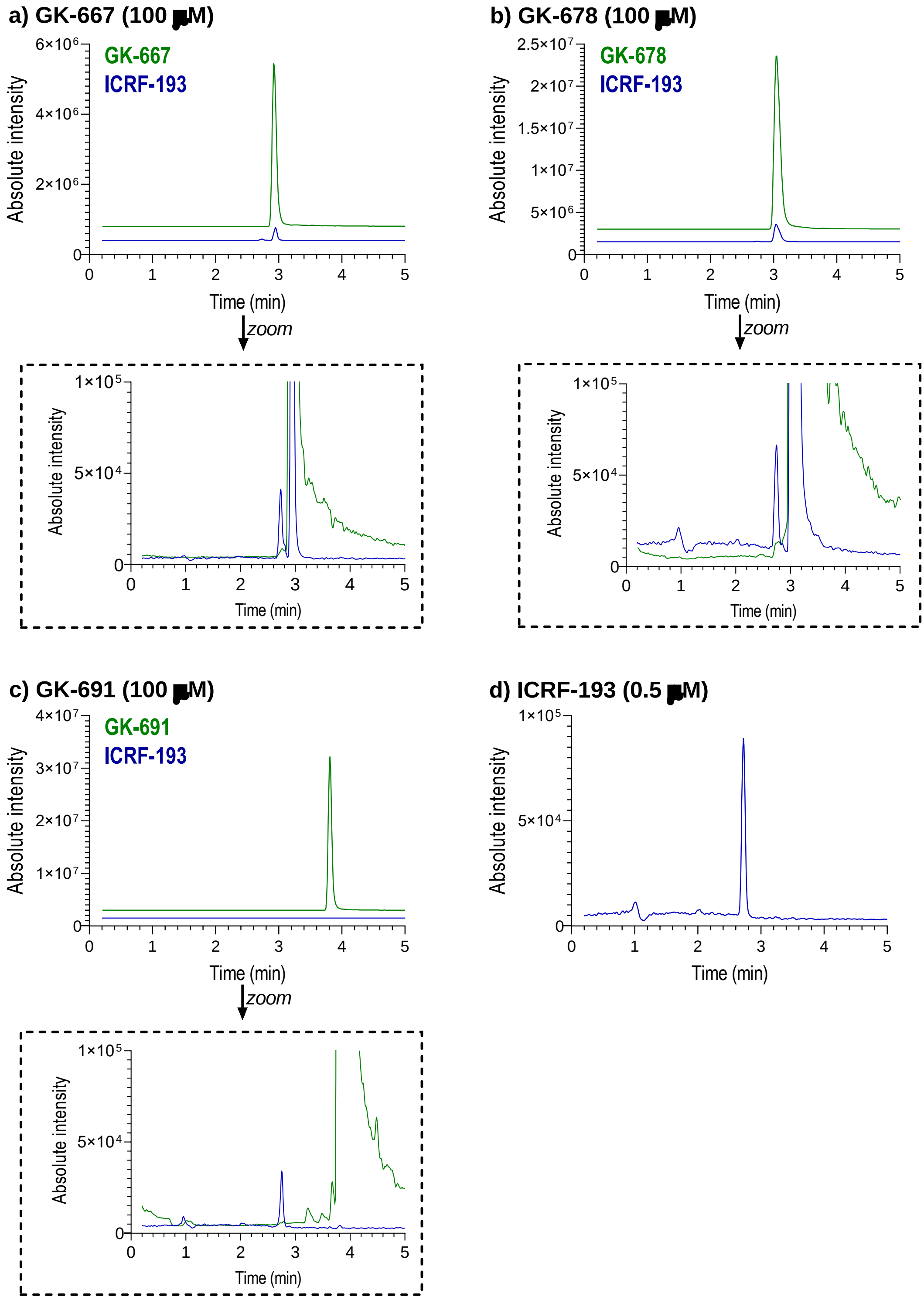

Tables

Table S1. Linearity, accuracy and precision for UHPLC-MS/MS assay of the analytes in **DMEM** (concentration range 0.2-100 μM, **method I.**).

Analyte	Concentration (μM)	Intra-day		Inter-day		ME	Linearity (weighted / r^2)
		Accuracy (%)	Precision (RSD)	Accuracy (%)	Precision (RSD)	I.S. normalized (RSD)	
GK-667	0.2	107.2	2.5	110.5	3.7	-	$1/x^2$ 0.999
	0.5	97.0	6.1	108.2	3.5	6.1	
	50.0	99.8	2.1	105.5	1.2	-	
	100.0	106.5	0.9	112.0	2.9	0.9	
ICRF-193	0.2	97.8	6.5	105.2	4.9	-	$1/x^2$ 0.999
	0.5	96.9	3.8	99.3	5.1	3.2	
	50.0	104.1	1.2	102.2	0.8	-	
	100.0	108.5	2.3	104.4	1.7	2.3	
ICRF-193$_{met}$	0.2	109.8	4.0	96.5	4.0	-	$1/x^2$ 0.999
	0.5	96.8	7.5	89.2	3.0	7.4	
	50.0	92.4	3.9	102.6	1.2	-	
	100.0	102.1	3.1	103.7	1.5	3.0	

ME – matrix effect

Table S2. Linearity, accuracy and precision for UHPLC-MS/MS assay of the analytes in **buffer** (concentration range 0.2-100 μM, **method I.**).

Analyte	Concentration (μM)	Intra-day Accuracy (%)	Intra-day Precision (RSD)	Inter-day Accuracy (%)	Inter-day Precision (RSD)	ME I.S. normalized (RSD)	Linearity (weighted / r^2)
GK-667	0.2	94.5	5.2	89.4	3.9	-	$1/x^2$ 0.990
	0.5	91.3	4.0	93.2	4.6	5.4	
	50.0	95.6	1.8	92.8	5.1	-	
	100.0	95.4	5.0	90.5	2.1	3.7	
ICRF-193	0.2	92.7	8.7	93.6	5.2	-	1/x 0.999
	0.5	90.2	5.0	105.7	2.0	6.4	
	50.0	98.4	2.5	92.7	1.2	-	
	100.0	101.9	0.5	92.7	1.3	0.9	
ICRF-193$_{met}$	0.2	95.1	2.1	85.7	3.3	-	1/x 0.999
	0.5	103.2	2.2	94.4	2.0	3.2	
	50.0	107.2	1.6	107.0	4.1	-	
	100.0	104.2	1.6	106.8	1.3	1.0	

ME – matrix effect

Table S3. Linearity, accuracy and precision for UHPLC-MS/MS assay of the analytes in **rabbit plasma** (concentration range 0.2-100 µM, **method I.**).

Analyte	Concentration (µM)	Intra-day Accuracy (%)	Intra-day Precision (RSD)	Inter-day Accuracy (%)	Inter-day Precision (RSD)	Recovery (%)	ME I.S. normalized (RSD)	Linearity (weighted / r^2)
GK-667	0.2	111.9	8.4	99.3	4.4	-	-	1/x 0.998
	0.5	104.6	8.7	101.3	6.3	-	4.0	
	50.0	105.3	4.0	107.2	5.7	92.6 ± 5.9	-	
	100.0	106.2	4.7	105.4	5.9	-	3.9	
ICRF-193	0.2	94.8	9.5	108.8	8.7	-	-	1/x 0.996
	0.5	91.0	6.4	99.4	3.6	-	4.8	
	50.0	90.3	3.3	97.5	2.8	94.2 ± 4.5	-	
	100.0	95.6	3.6	86.4	2.3	-	5.9	
ICRF-193$_{met}$	0.2	91.9	9.3	96.7	7.1	-	-	$1/x^2$ 0.995
	0.5	105.3	5.5	100.5	2.2	-	5.4	
	50.0	101.1	4.2	104.7	1.6	75.5 ± 9.5	-	
	100.0	92.0	6.3	91.9	1.4	-	4.7	

ME – matrix effect

Table S4. Linearity, accuracy and precision for UHPLC-MS/MS assay of the analytes **in neonatal rat cardiomyocytes** (concentration range 2.1-104.2 pmol/10^6 cells, **method I.**).

Analyte	Concentration (pmol/10^6cells)	Intra-day Accuracy (%)	Intra-day Precision (RSD)	Inter-day Accuracy (%)	Inter-day Precision (RSD)	Recovery (%)	ME I.S. normalized (RSD)	Linearity (weighted / r^2)
GK-667	2.1	91.8	2.7	99.2	2.5	-	-	1/x 0.999
	5.2	99.4	3.9	99.4	3.9	113.3 ± 4.9	3.0	
	52.1	110.3	0.6	100.5	5.4	-	-	
	104.2	103.3	1.7	99.3	11.3	-	8.5	
ICRF-193	2.1	97.0	9.7	101.1	11.1	-	-	1/x 0.999
	5.2	111.5	3.5	101.0	6.5	89.7 ± 6.7	5.0	
	52.1	98.7	2.5	103.1	2.6	-	-	
	104.2	99.9	1.3	101.7	2.7	-	5.9	
ICRF-193_{met}	2.1	115.2	3.8	111.1	2.9	-	-	unweighted 0.999
	5.2	109.3	5.8	105.0	8.5	55.7 ± 8.0	13.5	
	52.1	97.8	10.4	98.1	10.1	-	-	
	104.2	100.3	4.2	103.4	11.9	-	5.3	

ME – matrix effect

Table S5. Linearity, accuracy and precision for UHPLC-MS/MS assay of the analytes in **rabbit plasma** (concentration range 0.01-15.00 µM and 0.01-1.00 µM for ICRF-193 and ICRF-193$_{met}$, respectively, **method II**).

Analyte	Concentration (µM)	Intra-day Accuracy (%)	Intra-day Precision (RSD)	Inter-day Accuracy (%)	Inter-day Precision (RSD)	ME I.S. normalized (RSD)	Linearity (weighted / r^2)
ICRF-193	0.01	111.3	3.3	102.7	15.3	-	1/x 0.999
	0.05	100.2	5.8	96.9	10.4	6.8	
	0.50	96.8	8.0	95.8	1.4	-	
	10.00	100.4	5.8	92.9	11.4	11.4	
ICRF-193$_{met}$	0.01	98.1	10.6	105.3	7.9	-	1/x 0.997
	0.05	96.9	2.9	103.6	7.3	8.5	
	0.50	99.4	5.2	100.2	2.7	-	
	1.00	101.6	5.6	107.1	7.1	12.1	

ME – matrix effect

Table S6. Freeze and thaw stability of the ICRF-193 and ICRF-193met in the plasma at -80 °C and the room temperature. Data are shown as percentage of initial concentrations (n=3).

Compound	Initial concentration (µM)	% of initial concentration	
		1st freeze-thaw cycle	2nd freeze-thaw cycle
**ICRF-193**	0.05	98.8 ± 5.3	96.9 ± 8.2
	10.00	94.3 ± 2.3	89.6 ± 0.6
**ICRF-193met**	0.05	94.1 ± 4.3	91.7 ± 0.7
	1.00	93.9 ± 5.7	91.4 ± 7.0

Table S7. Stability of the extracted analytes in autosampler (8 °C). Data are shown as percentage of initial concentration (DMEM, buffer and plasma 100 µM and NVCMs 100 pmol/10^6 cells, n=3).

Time (h)	DMEM			Buffer		
	GK-667	ICRF-193	ICRF-193met	GK-667	ICRF-193	ICRF-193met
**0**	100.1	98.5	101.5	99.5	98.7	102.3
**8**	92.2	101.2	102.1	92.9	95.4	102.0
**10**	90.2	101.3	102.7	92.8	98.3	100.9
**Time (h)**	**NVCMs**			**Plasma**		
	**GK-667**	**ICRF-193**	**ICRF-193met**	**GK-667**	**ICRF-193**	**ICRF-193met**
**0**	103.3	100.0	100.0	101.5	99.8	100.4
**8**	105.2	107.6	97.7	102.1	96.4	96.5
**10**	103.3	108.4	95.0	102.7	93.8	99.1

Table S8. MS parameters setup.

**Method I**		**Method II**	
Nebulizing gas flow rate	3 L/min	Gas temperature	330 °C
Drying gas flow rate	15 L/min	Gas flow	12 L/min
Interface voltage	4.5 kV	Nebulizer	25 psi
DL temperature	250 °C	Sheat gas temperature	400 °C
Heat block temperature	400 °C	Sheat gas flow	9 L/min
CID gas pressure	230 kPa	Capillary	4500 V
		Noozle Voltage	2000 V

Table S9: The details on SRM used for quantitation of the analytes.

	Method I			Method II		
	Precursor ion (*m/z*)	Product ion (*m/z*)	Collision energy (eV)	Precursor ion (*m/z*)	Product ion (*m/z*)	Collision energy (V)
**ICRF-193$_{met}$**	319.1	187.2	-16	319.2	187.1<br>142.1<br>84.1	12<br>28<br>52
**ICRF-193**	283.3	169.1	-12	283.1	169.0<br>141.0<br>84.0	12<br>20<br>28
**GK-667**	457.4	141.2<br>169.3	-25<br>-33	*NA*	-	-
**GK-678**	485.0	169.1<br>141.1	-35<br>-28	*NA*	-	-
**GK-691**	637.1	169.0<br>190.0	-47<br>-33	*NA*	-	-
**I.S.$_{(A)}$**	347.0	201.0<br>142.0	-16<br>-31	347.2	201.1<br>142.0	12<br>32
**I.S.$_{(B)}$**	297.1	169.0<br>84.1	-14<br>-25	297.2	169.1<br>84.1	12<br>32

NA – not analyzed
